# Costs and cost-effectiveness of malaria control interventions - a systematic review

**DOI:** 10.1186/1475-2875-10-337

**Published:** 2011-11-03

**Authors:** Michael T White, Lesong Conteh, Richard Cibulskis, Azra C Ghani

**Affiliations:** 1 MRC Centre for Outbreak Analysis and Modelling, Department of Infectious Disease Epidemiology, Faculty of Medicine, Imperial College London, London, UK; 2 Centre for Health Policy, Institute of Global Health Innovation, Imperial College London, UK; 3 Global Malaria Programme, World Health Organization, Geneva, Switzerland

## Abstract

**Background:**

The control and elimination of malaria requires expanded coverage of and access to effective malaria control interventions such as insecticide-treated nets (ITNs), indoor residual spraying (IRS), intermittent preventive treatment (IPT), diagnostic testing and appropriate treatment. Decisions on how to scale up the coverage of these interventions need to be based on evidence of programme effectiveness, equity and cost-effectiveness.

**Methods:**

A systematic review of the published literature on the costs and cost-effectiveness of malaria interventions was undertaken. All costs and cost-effectiveness ratios were inflated to 2009 USD to allow comparison of the costs and benefits of several different interventions through various delivery channels, across different geographical regions and from varying costing perspectives.

**Results:**

Fifty-five studies of the costs and forty three studies of the cost-effectiveness of malaria interventions were identified, 78% of which were undertaken in sub-Saharan Africa, 18% in Asia and 4% in South America. The median financial cost of protecting one person for one year was $2.20 (range $0.88-$9.54) for ITNs, $6.70 (range $2.22-$12.85) for IRS, $0.60 (range $0.48-$1.08) for IPT in infants, $4.03 (range $1.25-$11.80) for IPT in children, and $2.06 (range $0.47-$3.36) for IPT in pregnant women. The median financial cost of diagnosing a case of malaria was $4.32 (range $0.34-$9.34). The median financial cost of treating an episode of uncomplicated malaria was $5.84 (range $2.36-$23.65) and the median financial cost of treating an episode of severe malaria was $30.26 (range $15.64-$137.87). Economies of scale were observed in the implementation of ITNs, IRS and IPT, with lower unit costs reported in studies with larger numbers of beneficiaries. From a provider perspective, the median incremental cost effectiveness ratio per disability adjusted life year averted was $27 (range $8.15-$110) for ITNs, $143 (range $135-$150) for IRS, and $24 (range $1.08-$44.24) for IPT.

**Conclusions:**

A transparent evidence base on the costs and cost-effectiveness of malaria control interventions is provided to inform rational resource allocation by donors and domestic health budgets and the selection of optimal packages of interventions by malaria control programmes.

## Background

Despite being a largely preventable and treatable disease, malaria is responsible for an estimated 800,000 deaths globally each year [1], with the majority of morbidity and mortality occurring in young children in sub-Saharan Africa. In addition to its impact on health, malaria imposes a heavy economic burden on individuals [2] and entire economies [3]. In response to calls for widespread control and elimination of malaria and the challenge of meeting the Millennium Development Goals, there has been a rapid scale-up of existing effective anti-malaria interventions, in particular insecticide-treated mosquito nets (ITNs) including long-lasting insecticidal nets (LLINs) [4-7], coupled with efforts to improve access to prompt and effective treatment [8,9]. There is a wide range of malaria control interventions whose efficacy and effectiveness have been repeatedly demonstrated over many years, including ITNs [10] and indoor residual spraying (IRS) [11], and interventions that have recently received increasing attention such as the use of artemisinin combination therapy (ACT) as first-line therapy [12], improved diagnosis using rapid diagnostic tests (RDTs) [13,14], and intermittent preventive treatment (IPT) [15-17]. In this review IPT is used as an umbrella term for intermittent preventive treatment in infants (IPTi), in children (IPTc), and in pregnant women (IPTp).

Having identified a range of interventions with proven efficacy, the challenge remains to scale-up their implementation in a sustainable, cost-effective and equitable manner. Decisions affecting the selection and coverage of interventions need to be taken in a rational, transparent manner using the best available evidence on efficacy, cost and cost-effectiveness. There have been a number of reviews looking at the efficacy and effectiveness of malaria interventions [10-12] and reviews of the costs and cost-effectiveness (CE) of selected interventions [18-20]. The seminal work of Goodman *et al *[21] reviewed and modelled the CE of a range of malaria interventions. Morel *et al *[22] made another significant contribution to the literature by using a model based on WHO CHOICE country estimates of general health care utilization costs [23] to estimate the cost-effectiveness of packages of malaria prevention and treatment interventions. However, there has not been a review of actual costs and CE of malaria treatment and prevention programmes in the last decade, despite the fact that these are likely to have changed with increasing economies of scale (the decrease in unit cost per intervention as the number of interventions delivered increases), and evolving market dynamics of the relatively new LLINs, ACT and RDTs. The need for such a review is timely given the changing global financial commitment to malaria [1,24] and the need for country-level decision making on which of the increasing number of tools have the greatest impact on reducing malaria with the minimum cost and are therefore the most efficient use of resources.

A systematic review of the published literature on the cost and cost-effectiveness of malaria control interventions published from 2000-2010 is presented. Studies published in this time period were implemented from 1990-2010. Unlike other reviews, which are generally country-specific [25] or focus on a single intervention strategy [18,26], the costs and CE of interventions over time and across world regions are presented and compared. All data is synthesized to allow cross-comparison between different interventions and study locations, and the full details extracted from each identified study are made available as Supplementary Online Material.

## Methods

### Review of cost and cost-effectiveness studies

A systematic search of the published English-language literature on studies of the cost of all malaria interventions published between 2000 and 2010 was conducted using the electronic online database PubMed. These studies were implemented between 1990 and 2010. The MESH term used was '(malaria OR falciparum) AND cost'. This was supplemented by searches of Google Scholar and African Journals Online using the same MESH term as well as iterative reviews of the reference lists of relevant published papers and searches of the grey literature consisting of PhD theses and reports identified in references, reports to WHO from consultation projects for the evaluation of the costs of ITN distribution and additional searches on the Social Science Research Network and the Bath Information Data System. In addition, searches for costing studies of *Plasmodium vivax*, *Plasmodium ovale *and *Plasmodium malariae *were undertaken. All the abstracts from the identified publications were reviewed and the publications selected for review if they contained primary data on the cost of one or more malaria interventions.

A proportion of the studies identified in the review of the costs of malaria interventions contained estimates of cost-effectiveness. These studies were supplemented by an additional systematic search of the cost-effectiveness literature to identify those studies providing cost-effectiveness estimates but not primary cost estimates. The MESH terms used were: '(malaria OR falciparum) AND (cost OR effective OR effectiveness OR benefit)'. As only thirty three published studies of cost-effectiveness were identified in the period 2000-2010, the search was extended to consider studies published in the period 1990-2010. These searches were further supplemented by iterative reviews of the reference lists of relevant published papers and searches of the grey literature consisting of PhD theses and reports. Abstracts and full publications were reviewed and included if they contained estimates of the cost-effectiveness of interventions in terms of health outcomes.

**Figure 1:**
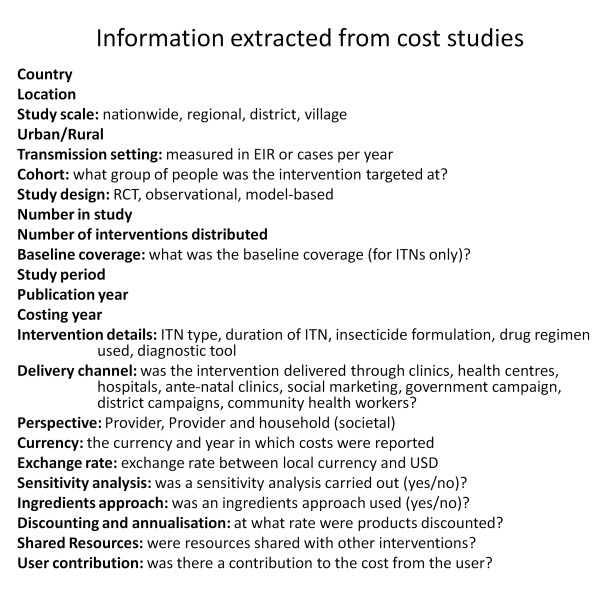
**Information extracted from cost studies**.

The selected studies were stratified into six categories according to intervention: ITNs, IRS, IPT, diagnostics, treatment and other. For each study the information in Figure 1 was extracted where available. Where possible, both the financial and economic costs were extracted. Where an ingredients approach to costing was used, the cost was split into the following categories: 'nets', 'insecticide', 'diagnostics', 'treatment', 'personnel', 'training', 'IEC', 'distribution', 'transport', 'storage', 'overheads', 'capital', 'study costs', 'other' and 'user'. Where studies reported the costs for multiple outcomes, e.g. cost per ITN distributed or cost per person protected with ITNs, the cost for each outcome was extracted.

Where reported, the results of sensitivity analyses were extracted from the studies. In some studies the sensitivity analyses are 95% confidence intervals when intervention parameters and costs are varied simultaneously according to some distribution. However, most studies, and in particular those published before the wide-scale use of probabilistic sensitivity analysis, only include a simple one-way sensitivity analysis where one parameter at a time is varied. In these studies the limits of the sensitivity analyses were taken to be the highest and lowest estimates of costs or cost-effectiveness ratios.

All of the extracted information can be found in Additional File 1 'CostReview.xls'.

### Costs

Financial and economic total costs are presented if both were identified in the original paper. Financial costs reflect the unit cost of an intervention and the resources required for its delivery in terms of the actual expenditures incurred. The economic costs capture the opportunity cost of all resources used to provide an intervention, whether or not they incur a financial expenditure. For example, the time health personnel are involved in treating malaria often represents an economic cost, because they are already receiving a salary for the broad range of services they provide and, while they do not receive additional funding for the specific malaria intervention being assessed, they could have spent their time on other activities. Alternatively, if drugs have been donated or community volunteers are helping for 'free', an economic cost will attach a market value to these resources. Although economic costs provide a better of measure of the total costs associated with distributing interventions, a much larger proportion of interventions reported financial costs, and hence financial costs are focussed on in this review. Costs are commonly collected from two different perspectives: provider and societal. The first relates to the costs, usually borne by the public health system, of providing preventive or treatment strategies. The second perspective, societal, refers to the wider direct and indirect costs not only to the provider but also to the household in terms of their lost time and income.

Once delivered, preventive interventions such as ITNs, IRS and IPT will provide protection against malaria for a number of months or years. The duration of protection offered by an intervention will have cost implications as it will determine when the intervention next needs to be delivered. For example, if IRS provides protection for six months, then spraying will be required twice yearly to provide constant protection. To allow greater comparability between interventions the standardized annual costs of protecting one person for a full year with ITNs, IRS or IPT was calculated. Standardization was not undertaken for diagnosis and treatment since the annual cost of these interventions depends on local incidence of malaria.

The cost per treated net year (TNY) captures the cost of ensuring one person is protected by an appropriately treated bed net for one year. The standardized cost of protection with ITNs was taken to be the cost per TNY for those studies reporting TNYs. Where TNYs were not reported, the standardized cost was taken to be the financial cost divided by the lifetime of the ITN. When the lifetime of the ITN was not reported, a three-year lifetime was assumed. Financial, and not economic, costs are more often discussed in light of the greater number of studies that presented their findings as financial costs. Some costing studies of IRS provided estimates of the cost of protection per person per year, while some provided estimates of the cost of a spray round per person. Where the cost of protection per person per year was not estimated, a standardized financial cost of protection per year was calculated by assuming two spray rounds were necessary for a full year of protection. This method does not account for the seasonal nature of malaria transmission and the additional benefit obtained by spraying before the rainy season. The standardized cost of a year's protection with IPT was calculated by extrapolating to the number of courses required for a year's protection. In the case of IPTp, it is assumed that there is only one pregnancy per year, and hence the cost of a year's protection is equal to that of protection throughout pregnancy. In the case of seasonal administration of IPT to school-age children, it was assumed that protection during the rainy season provided protection throughout the year and hence the standardized annual cost was assumed equal to the unit financial cost.

When reporting the cost of an intervention from a number of studies the median and range is given instead of the mean as this prevents the summary measure of cost being skewed by outliers. When multiple years of costing were carried out, data from the most recent year was taken.

### Inflation of costs

The costs of the interventions surveyed in this review were extracted from studies evaluating costs in a range of international currencies over a period of time dating back to 1990. In order for meaningful comparisons between costs from different studies to be made, costs were adjusted to a common year and a single currency. Costs of interventions are first converted from local currency to US dollars using the exchange rate at the year of costing and then inflated to 2009 USD using USD inflation rates. There are alternative methods for inflating the costs of interventions [27], see Appendix 1 for more details.

Throughout, the years reported in the results section refer to the year the costs were collected and not the year they were published.

### Effects

Only studies reporting cost-effectiveness ratios against endpoints relevant to health-related malaria outcomes were included in the comparison of cost-effectiveness outcomes. Thus, studies calculating incremental cost-effectiveness ratios (ICERs) per DALY averted, malaria-associated deaths averted or malaria cases averted were included. Studies calculating intermediate outcomes, such as ICERs per ITN distributed or house sprayed were excluded as these did not directly relate to a health outcome, making generalization and comparison with other studies more difficult. There was substantial variation in the definition of a case of malaria between studies, ranging from parasitaemia to clinical or severe episodes of malaria, to inclusion of co-morbidities such as anaemia. The definition of a case of malaria will significantly influence the reported cost-effectiveness. For example, if the incidence of uncomplicated clinical malaria is higher than the incidence of severe malaria then it will be cheaper to avert an uncomplicated case than a case of severe malaria. There are substantial problems associated with accurately determining the number of cases of malaria or deaths averted by an intervention. The impact on CE of variation in the number of reported cases of malaria will be partially addressed by sensitivity analyses, at least in those studies where they are carried out. Cost-effectiveness studies do not all have the same 'starting point'. It is important to note when interpreting the effects of an intervention that the baseline packages of interventions already available can vary considerably across studies and this is not always made explicit. For example, the cost effectiveness of an intervention is likely to appear much more favourable in a setting where very few other malaria strategies are in place, whereas the impact of the same intervention is likely to have a less marked effect, and hence appear less cost effective, in a setting where ITN coverage is high and widespread, prompt and effective treatment is on offer. In addition cost effectiveness will depend on the intensity of malaria transmission and the behavioural characteristics of the local vector population.

## Results

### Costs of malaria interventions

**Figure 2:**
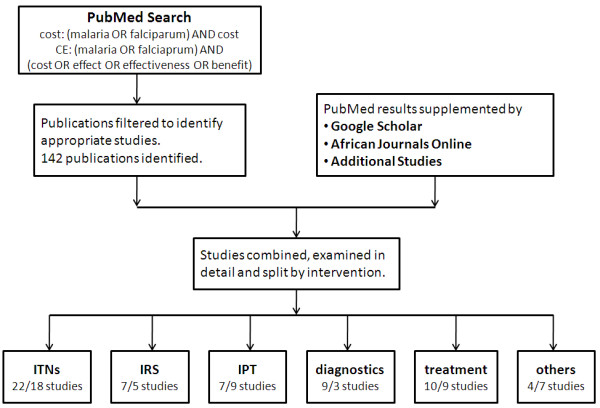
**Search strategy for costing studies**. The number of cost and cost-effectiveness studies identified in each category is shown (left-hand number is cost/right-hand number is cost-effectiveness (CE)).

**Figure 3:**
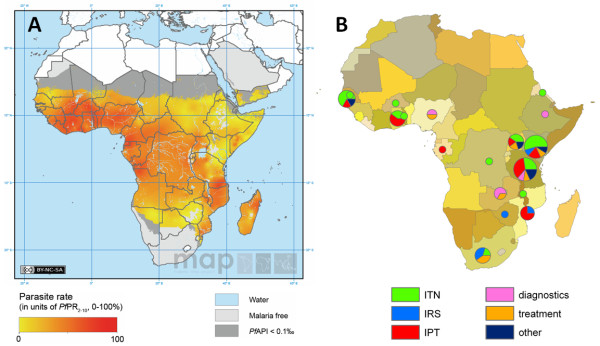
**Geographical location of costing and cost-effectiveness studies**. A: Map of *P. falciparum *parasite prevalence in Africa estimated in 2007 from the Malaria Atlas Project [27]. B: Geographical location of African based studies of cost and cost-effectiveness. 78% of reviewed studies were located in Africa, 18% in Asia and 4% in South America.

**Figure 4:**
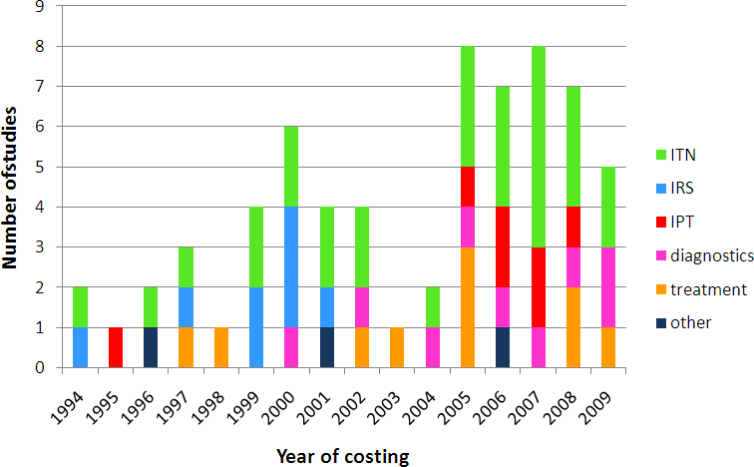
**Year of costing of cost and cost-effectiveness studies**. The year refers to when the intervention was costed and not the publication year.

In total, fifty five relevant costing studies were identified, thirty three of which also included estimates of cost-effectiveness. An additional ten studies reporting cost-effectiveness estimates but no primary cost data were identified resulting in a total of forty three relevant cost-effectiveness studies (Figure 2). The interventions studied included ITNs, IRS, IPTi/c/p, vaccines, malaria diagnostics, treatment of uncomplicated malaria, treatment of severe malaria in health centres or hospitals, larviciding, larvivorous fish, malaria early warning systems, environmental management, drug treatment, rapid diagnostics, and combined prevention and treatment programmes. There was a great deal of heterogeneity in the type of costing study identified: randomized controlled trials of intervention efficacy including detailed costing information; large scale intervention programmes implemented on a regional or national level, involving estimates of costs extrapolated from data collected from health officials; and model-based studies combining data from different sources to produce estimates of cost. Overall the studies were identified from countries throughout malaria endemic areas of the world, but with a heavy focus on Sub-Saharan Africa (78% of studies). Within Africa, most were conducted in East Africa, in particular Kenya and Tanzania, and The Gambia in West Africa. A geographical comparison of study locations and the burden of *P. falciparum *malaria [28] is shown in Figure 3. There are many countries with a high burden of malaria but little or no studies of the cost or cost-effectiveness of malaria interventions. A large proportion of the studies were undertaken between 2005-2010, many of these relating to new interventions, in particular IPTi/c/p (Figure 4).

### Insecticide-treated nets

Twenty-two studies of the costs of distributing ITNs were identified, ten of which were for the distribution of LLINs (Additional file 2: Table S1). The studies measured the cost of a number of different outcomes: (i) cost per net distributed; (ii) cost of distribution only (excluding the cost of net purchase); (iii) cost per treated net year (TNY), i.e. year of effective protection; and (iv) cost per person protected. These outcomes differ in a number of ways. There is variation in how studies include insecticide re-treatment. In some studies, conventional ITNs were distributed pre-treated with insecticide, in others ITNs are distributed with sachets of insecticide for treatment by the users, and other studies include the cost of re-treatment some time after distribution. The cost per TNY captures the added costs and benefits associated with insecticide re-treatment, although most studies preferred to present cost per net distributed. The recent shift to distribution of LLINs instead of conventional ITNs has made comparison of costs easier as the additional costs associated with insecticide re-treatment are avoided.

The cost per ITN distributed is typically higher than the cost per person protected by a net, as it is usually assumed that nets are shared (e.g. mother and child). However some studies estimate the cost of delivering an ITN to a target group, such as newborn infants, in which case the cost per person protected in the target group may be more than the cost per ITN delivered (e.g. to ensure infants are covered by nets more than one net may need to be distributed per infant, as some nets may be used by other family members.).

**Figure 5:**
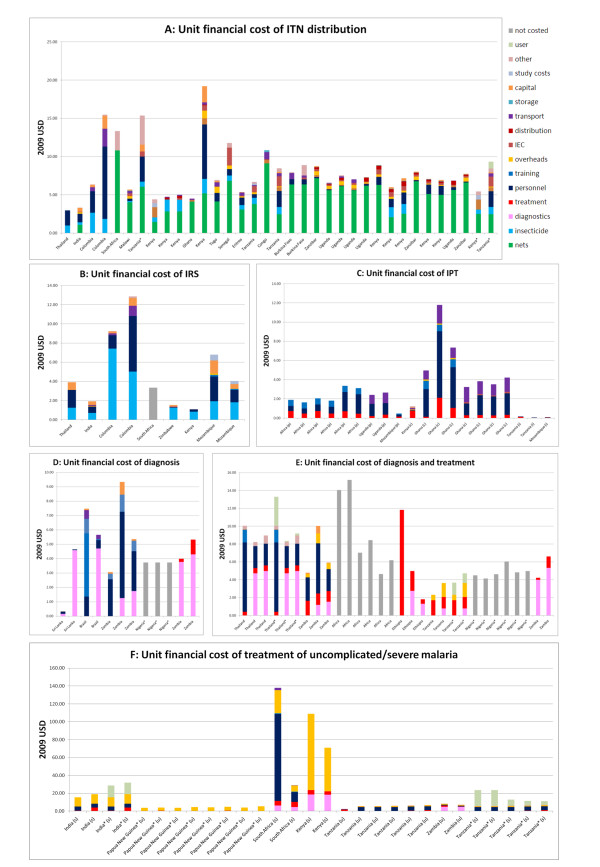
**Unit financial costs of malaria control interventions broken down into components**. A: Unit financial cost per ITN distributed. Data is taken from Additional file 2: Table S1. B: Unit financial cost per person protected by IRS. Data is taken from Additional file 3: Table S2. C: Unit financial cost per course of IPT. (i) indicates IPT in infants, (c) IPT in children, and (p) IPT in pregnant women. Data is taken from Additional file 4: Table S3. D: Financial cost of diagnosing a patient for malaria. Data is taken from Additional file 5: Table S4. E: Financial cost of diagnosis and treatment with ACT. Data is taken from Additional file 5: Table S4. F: Financial cost of treatment of either uncomplicated or severe malaria. (u) indicates uncomplicated malaria and (s) severe malaria. Data is taken from Additional file 6: Table S5. All studies were costed from a provider perspective except those marked * which were costed from a societal perspective. All costs are in 2009 USD.

The median financial cost per ITN distributed (in the first year) was $7.03 ranging from $2.97-$19.20 whilst the median economic cost (across the expected lifetime of the net) was $4.15 ranging from $2.97-$10.05. The median standardized financial cost for a year's protection with ITNs was $2.20 ranging from $0.88-$9.54. Across the studies that reported a detailed breakdown of costs, a mean proportion of 63% (range 12%-92%) of the cost of distributing a net was attributable to nets and insecticide, 17% (range 2%-67%) was attributable to personnel and training, and 7% (range 1%-17%) to IEC and transport (Figure 5).

### Indoor residual spraying

Seven studies of the costs of spraying houses with indoor residual insecticide were identified (Additional file 3: Table S2). Two different outcomes were measured across the studies: (i) cost per person protected, and (ii) cost per dwelling sprayed. All studies estimated the cost per person protected and four studies estimated the cost per dwelling sprayed. There was much variation in the seven studies identified with locations from Africa, Asia and South America, different insecticide classes and different periods of protection. The IRS studies were all relatively old - the most recent year of costing was 2001 (Figure 4). The number of spray rounds and the timeframe of protection varied between studies, as such reported financial and economic costs cannot be directly compared between studies. Direct comparisons can be made between standardized financial costs which have been adjusted to have a timeframe for protection of one year. The median financial cost per person protected by IRS was $3.91 ranging from $1.11-$12.87 whilst the median economic cost was $3.41 ranging from $1.14-$6.23. The median standardized cost of a year's protection with IRS was $6.70 ranging from $2.22-$12.85. The relationship between cost per person protected and cost per house sprayed varied depending on the number of people per house, which varied from 1.6 to 5.4. Several different insecticide formulations were used in the studies: DDT and the pyrethroids, deltamethrin and lambdacyhalothrin. The mean proportion of the cost attributable to the cost of insecticide was 49% (range 29%-81%), and the mean proportion attributable to personnel and training was 34% (range 4%-48%) (Figure 5).

### Intermittent preventive treatment

Eight studies of the costs of administering a course of intermittent preventive treatment were identified: two of these studies were for IPT in infants (IPTi), three for IPT in children (IPTc), and three for IPT in pregnant women (IPTp) (Additional file 4: Table S3). The median financial cost of protection was $0.10 (range $0.08-$0.18) for IPT in infants, $4.03 (range $1.25-$11.80) for IPT in children, and $2.06 (range $0.47-$3.36) for IPT in pregnant women. Full treatment with IPTp is assumed to confer protection for a timeframe of one year (assuming no more than one pregnancy per year); full treatment with IPTc is assumed to confer protection for a timeframe of one year; and a dose of IPTi is assumed to confer protection for two months. As studies reported the costs of administering IPT with varying number of courses and doses, the standardized cost of a year's protection was also calculated. The median cost of a year's protection was $0.60 (range $0.48-$1.08) for IPTi, $4.03 (range $1.25-$11.80) for IPTc, and $2.06 (range $0.47-$3.36) for IPTp. Personnel and training accounted for the majority of the costs of administering a course of IPTi and IPTp, as the drug used (sulphadoxine pyrimethamine) was relatively inexpensive. The median cost of administering IPT to infants and pregnant women was substantially less expensive than the costs of administration to children as the drugs can be delivered to infants alongside vaccinations in the well-established Expanded Programme on Immunization and to pregnant women through existing ANC clinics. For IPTi on average 48% (range 14%-71%) of the cost of a course of treatment was attributable to the cost of drugs, 37% (range 24%-56%) to personnel and training, and 15% (range 5%-40%) to transport and IEC. For IPTc on average 13% (range 8%-60%) of the cost of a course of treatment was attributable to the cost of drugs, 60% (range 47%-77%) to personnel and training, and 25% (range 13%-49%) to transport and IEC. For IPTp on average 22% (range 9%-39%) of the cost of a course of treatment was attributable to the cost of drugs, 67% (range 45%-79%) to personnel and training, and 11% (range 0%-42%) to transport and IEC (Figure 5).

### Diagnosis and treatment

Nine studies of the costs of malaria diagnosis were identified (Additional file 5: Table S4). The diagnostic tools used were clinical observation of symptoms, detection of parasites by microscopy or rapid diagnostic tests (RDTs). The studies measured combinations of the following outcomes: (i) cost per patient diagnosed (seven studies); and (ii) cost per patient diagnosed and treated (six studies). The financial costs per patient diagnosed, and per patient diagnosed and treated are shown in Additional file 5: Table S4. All studies comparing the cost of *P. falciparum *diagnosis (but not the cost of treatment) by RDTs and microscopy found RDTs to be more cost-effective. A Sri Lankan study [29] comparing the cost of *P. vivax *by RDT and microscopy found microscopy to be most cost-effective, a finding attributable to the high cost of the immunochromatographic test for *P. vivax*. There were two studies comparing the costs of diagnosis and treatment with RDTs and microscopy: one study found RDTs to be less expensive [30] while the other found microscopy to be more cost-effective [31]. Two studies compared the cost of RDT diagnosis and ACT treatment with presumptive ACT treatment; one found RDT diagnosis to be more cost-effective [32], whereas the other found presumptive treatment to be more cost-effective [33]. These apparently conflicting results are due to differences in where diagnosis occurs and diagnostic throughput, and hence may not be generalise to other settings. Diagnosis by microscopy will be inexpensive in a well-established health centre or hospital with trained microscopists, whereas RDTs will be less expensive in more inaccessible rural areas. The cost of diagnosis and treatment will also be dependent on parasite prevalence. On average 39% (range 13%-99%) of the cost of diagnosing a case of malaria was attributable to the diagnostic technique and 42% (range 1%-83%) to personnel and training. For studies of diagnosis and treatment, 27% (range 12%-95%) of the cost was attributable to diagnosis, 41% (range 5%-47%) to treatment, and 17% (range 0%-56%) to personnel and training (Figure 5).

### Treatment of uncomplicated and severe malaria

Patients with episodes of uncomplicated malaria can be treated at home by community health workers, at health facilities or as hospital outpatients. In addition to those studies on diagnosing and treating uncomplicated malaria identified in the section on diagnosis, five studies of the costs of treating uncomplicated episodes of malaria were identified (Additional file 6: Table S5). Three studies estimated the cost of hospital treatment from the provider's perspective and two studies estimated the cost from a societal perspective. All studies estimated either the financial or economic cost of treating an episode of malaria with a number of drugs including artemisinin combination therapy, sulphadoxine-pyrimethamine, chloroquine and quinine. The median financial cost of treating an episode of uncomplicated malaria (either as hospital outpatients or at health centres) was $5.84 (range $2.36-$23.65), and the median economic cost was $22.48 (range $9.14-$37.99).

Patients with severe episodes of malaria usually need to be treated as hospital inpatients to ensure effective treatment. Six studies of the cost of treating severe episodes using different treatment regimens of malaria requiring hospitalization were identified (Additional file 6: Table S5). All studies estimated the cost of hospital treatment from the provider's perspective and one study also estimated the cost from a societal perspective. There was substantial variation between the cost per patient treated due to differences in study location, type of health facility, disease severity, and treatment regime. The median financial cost of treating a hospital inpatient was $30.26 (range $15.64-$137.87) and the economic cost was $64.50 (range $26.99-$288.79).

For patients with uncomplicated malaria, the mean proportion of costs attributable to personnel and training was 29% (range 10%-78%), 11% (range 1%-80%) to treatment and diagnostics, 24% (range 3%-98%) to overheads and 31% (range 0%-75%) was borne by the patient. For patients with severe malaria the mean proportion of financial costs attributable to hospital overheads was 47% (range 19%-79%), 35% (range 4%-71%) to personnel and training, and 17% (range 2%-36%) to treatment and diagnostics.

### Effect of scale of study on estimates of cost

**Figure 6:**
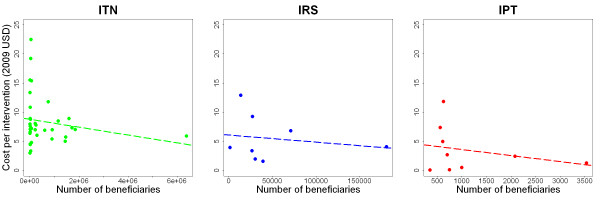
**Economies of scale for ITNs, IRS and IPT**. Financial costs per beneficiary of malaria control interventions, as a function of the number of beneficiaries of the program or project evaluated. Dotted lines represent straight lines of best fit.

There was great variation in the scale of the intervention programmes and projects analysed in the studies reviewed: some evaluated the cost of implementing an intervention based on less than 100 beneficiaries or patients, while other studies estimated the cost of implementing an intervention to more than 100,000 beneficiaries. Economies of scale may result in cost savings per unit when an intervention is widely implemented. Figure 6 shows the relation between cost per beneficiary and scale of the studies of malaria control interventions (as measured by the number of beneficiaries). For ITN distribution (green), there is a trend towards lower distribution costs for larger numbers of beneficiaries, but this trend is not statistically significant (linear regression P value = 0.29). In addition there is much less variation in studies with a larger number of beneficiaries. There is a trend for lower costs of implementing IRS in studies with a larger number of beneficiaries (blue), although this is not significant (linear regression P value = 0.68). For IPT (red), the cost per course administered decreases as the number of beneficiaries is increased, but again this is not statistically significant (linear regression P value = 0.48).

### Cost-effectiveness of malaria interventions

**Figure 7:**
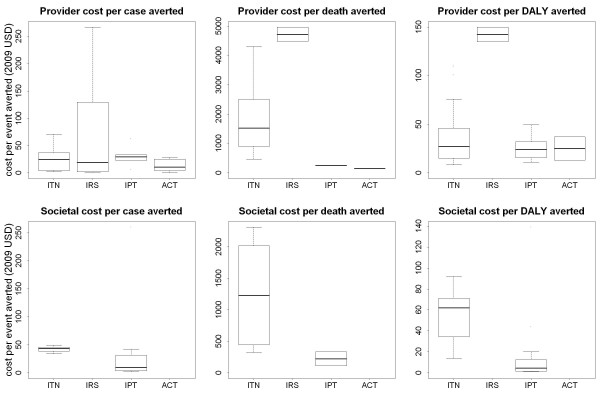
**Cost-effectiveness of anti-malarial interventions against deaths averted, DALYs averted and cases of malaria averted**. The ICERs for ITN, IRS and IPT are against a baseline of no widespread preventive interventions. The ICERs for ACT are calculated against a baseline of alternative treatment strategies, and hence care must be taken when comparing ICERs for preventive and treatment based interventions.

In total, forty eight studies were identified that considered the cost-effectiveness of malaria interventions including ITNs, IRS, IPT, vaccines, environmental management, drug treatment, diagnostics and combined treatment and prevention. The full results are presented in Additional file 7: Table S6. The majority of studies used a provider perspective, with a small number including a societal perspective. Figure 7 shows the distribution of the published estimates of the incremental cost-effectiveness ratios (ICERs) of four interventions against three different endpoints: cost per case of malaria averted, cost per death averted and cost per DALY averted.

From a provider perspective, the median incremental cost effectiveness ratio per DALY averted was $27 (range $8.15-$110) for ITNs, $143 (range $135-$150) for IRS, and $24 (range $1.08-$44.24) for IPT. Despite large variation in delivery costs between studies and settings, all of the major preventive interventions (ITNs, IRS, and IPT for infants, children or pregnant women) and ACT treatment were consistently cost-effective against a threshold of $150 per DALY averted ($260 at 2009 prices) [34,35]. IPTi and IPTp were found to be the most cost-effective preventive interventions against all endpoints considered. The superior cost-effectiveness of IPT was limited to the target groups of infants and pregnant women, with the CE of IPT in children being comparable with the CE of ITNs and IRS. Based on the evidence of this review, it was not possible to determine conclusively whether ITNs or IRS were more cost-effective. However, the results of studies comparing both interventions at the same site by Bhatia *et al *[36], Kamolratanakul *et al *[37] and Goodman *et al *[38] indicate that ITNs are more cost-effectiveness than IRS, in contrast to the finding by Guyatt *et al *[39] that IRS was more cost-effective than ITNs in response to epidemic malaria. All studies identified found effective treatment of episodes of uncomplicated or severe malaria with ACT to be highly cost-effective when compared to other anti-malarial drugs.

One caveat when interpreting the results from Additional file 7: Table S6 is that the ICERs are calculated from varying perspectives. In addition, with the exception of the vaccine studies by Tediosi *et al *[40,41] and an ITN study by Wiseman *et al *[42], which explicitly include mass effects, all studies considered were designed to evaluate the benefit of an intervention at the individual level. ITNs and IRS are likely to have substantial additional benefits when applied on a large scale since they can reduce the size of the vector population, potentially reducing transmission, as well as providing protection to individuals receiving the intervention. Whilst these effects are captured in estimates of impact of ITNs or IRS on morbidity outcomes from community randomized trials, their true effect will vary by transmission setting and by coverage level and hence cannot easily be extrapolated from one setting to another.

## Discussion

A transparent evidence base on the costs and cost-effectiveness of malaria control interventions is provided, to inform resource allocation by international and domestic financers of health programmes, and the selection of optimal packages of interventions for malaria control programme managers. The median financial cost per ITN distributed was $7.03 (range $2.97-$19.20), $3.91 (range $1.11-$12.87) per household for IRS, $0.10 (range $0.08-$0.18) for IPT in infants, $4.03 (range $1.25-$11.80) for IPT in children, and $2.06 (range $0.47-$3.36) for IPT in pregnant women. The median financial cost of diagnosing a case of malaria was $4.32 (range £0.34-$9.34). The median financial cost of treating an episode of uncomplicated malaria was $5.84 (range $2.36-$23.65) and the median financial cost of treating an episode of severe malaria was $30.26 (range $15.64-$137.87). The wide ranges in the estimates of unit costs represent different durations of protection, and are a consequence of the wide variation in the type of costing study reviewed.

One of the key drawbacks of costing studies is that they are often not undertaken alongside an evaluation of the clinical and epidemiological effect of the intervention under investigation. Thus it will cost the same to distribute a bed net in an area of high transmission as in an area of low transmission. Cost-effectiveness analyses incorporate information on both intervention costs and the impact on health. All of the major preventive interventions and ACT treatment were consistently cost-effective against a threshold of $150 per DALY averted ($260 at 2009 prices) [34,35].

Care must be taken when comparing the cost-effectiveness of prevention and treatment-based interventions, as the denominator populations at risk may not be directly comparable due to differences in age, location, or exposure to malaria. Preventive interventions are administered to individuals before future disease status is known, (e.g. an ITN may be delivered to a person who would not have become infected anyway) whereas treatment with ACT is administered to an individual conditional on them experiencing an episode of malaria and coming into contact with a health facility where a study is being undertaken. In studies of the cost-effectiveness of preventive interventions, comparisons will often be made between a population receiving the intervention and a control population not receiving the intervention. Such a study design is more difficult for treatment-based interventions which must always compare the treatment under investigation with an alternative treatment. These highlighted difficulties make direct comparison of the cost-effectiveness of prevention and treatment-based interventions difficult. In addition, the cost of diagnosis and treatment programmes may increase if active case detection is undertaken (searching for cases) rather than passive. Although treatment and prevention-based intervention will often be competing for the same donor funds, they should be seen as complementary and not in direct competition for resources.

The primary studies of costing data identified estimated the costs of single interventions in the absence of other anti-malaria interventions, with the exception of a study by Picard *et al *[43]. However estimates of the costs and cost-effectiveness of combined interventions were possible in model-based studies [21,22]. Given the renewed enthusiasm for large-scale malaria control and elimination efforts, control programmes based on multiple interventions are becomingly increasingly common [44-46]. Anti-malaria interventions will increasingly be deployed as part of wider health system strengthening packages leading to possible economies of scope: witness the IPTi studies by Manzi *et al *[47] where the cost of a course of intermittent preventive treatment was reduced due to its administration alongside the already existing (and therefore not an additional financial cost) Expanded Programme on Immunization. Programme donors such as The Global Fund and GAVI are committed to supporting linkages between malaria control and strengthening of maternal, neonatal and child health through harmonized funding platforms [48,49]. As such it may be misleading to consider the costs of malaria control in isolation.

The cost-effectiveness literature is also lacking in the evaluation of combined malaria interventions. Apart from the studies by Akhavan *et al *[25] and Mills [50] on the evaluation of national or district level malaria control programmes, only one study considers simultaneously deployed interventions. Picard *et al *[43] compared the cost-effectiveness of ITNs and ITNs with chemoprophylaxis and found that ITNs alone were more cost-effective for averting malaria-associated deaths, but ITNs with chemoprophylaxis were more cost-effective for averting cases of malaria. Counter-intuitive results such as this are not immediately obvious from separate studies of ITNs and ITNs with chemoprophylaxis.

In the absence of detailed studies on the evaluation of the costs and cost-effectiveness of combined interventions, costing models can provide invaluable information for individuals implementing malaria control programmes. Two such models stand out: (i) a decision tree model by Goodman *et al *[21], building on an extensive review of the cost-effectiveness literature [19], estimated the cost-effectiveness of ITNs, IRS, chemoprophylaxis of children, antenatal care and improvement of case management; and (ii) a costing and cost-effectiveness model by Morel *et al *[22] based on data from the literature and prices from the WHO-CHOICE database [51]. This study estimated the cost-effectiveness of several combinations of anti-malaria interventions (ACT, SP, CQ, ITNs, IRS and IPT) in East and West African settings.

An important drawback of these models is that they do not account for the dynamics of malaria transmission: model predictions of health impact assume a fixed number of cases or deaths averted per unit of service/output, so the additional benefit of mass effects and their impact on the vector population (or, conversely, possible saturation and overlap of interventions leading to diminishing returns) at higher coverage levels are ignored. One way to overcome these difficulties is by incorporating cost-effectiveness evaluation into models of the transmission dynamics of malaria. Tediosi *et al *[41,52] and Smith *et al *[53] have developed a transmission model of the clinical epidemiology and natural history of *P. falciparum *for the evaluation of the cost-effectiveness of an infection-blocking malaria vaccine, and Ross *et al *have extended this model to intermittent treatment [54]. This model captures the benefit of herd immunity and allows cost-effectiveness to be estimated across a range of transmission settings. These types of models are ideally suited for evaluating the costs and cost-effectiveness of combined malaria interventions.

In recent years there has been an encouraging increase in the number of studies investigating the cost and cost-effectiveness of key malaria control interventions (Figure 4). The costing methodology used in published studies has significantly improved, with studies increasingly taking a detailed ingredients approach to costing following the recommendations of Creese and Parker [55] and Kolaczinski *et al *[18]. More detailed methodologies allow greater comparability between studies and allow the results to be generalised/extrapolated to other settings. For example, if there is a known difference between sites in the price of a net or insecticide but the costs accruing to the supply chain can be assumed to be the same across settings, then the cost of distributing an ITN can still be estimated. One key area for improvement is the need for more data collection in countries with a high malaria burden and large populations as existing studies were clustered around a few well-recognised sites in Tanzania, Kenya and The Gambia (Figure 3).

All costs in this review have been presented in 2009 USD to allow easy comparison between studies. However, focusing on a single value to represent the cost of implementing a malaria intervention can conceal a great deal of variation in the methodology used to arrive at that figure. Final costs can depend on the choice of costing components, the method used for inflation, scale and scope of implementation, as well as local study factors. Sensitivity analyses go some way towards capturing this variation in costs. In the past decade, sensitivity analyses have become increasingly sophisticated, developing from simple one-way analyses where one costing component at a time is varied to probabilistic sensitivity analyses where Monte Carlo methods are used to vary multiple costing components simultaneously and produce a distribution of possible costs or cost-effectiveness ratios. The variation in costs may also depend on the scale of the study; larger studies may have accurate estimates of total costs, but lack the detailed record-keeping that is possible in smaller studies in more controlled environments; whereas the costs in smaller studies can be affected by start-up and monitoring expenses.

There is still room to improve transparency and consistency when reporting the assumptions, methodologies and findings of economic evaluations to allow for greater comparisons across interventions and thus help decide the most appropriate strategies of treatment and prevention. On a more positive note, however, increasing cost and cost-effectiveness studies (both in terms of their frequency and scale), improved modelling techniques that enable us to extrapolate from small-scale studies to larger populations, and a recognition of the importance of exploring variation and uncertainty associated with costs as well as effects, has led to great opportunities for economic evaluations to contribute to the debate on which malaria interventions should be deployed, and where, to achieve optimal heath gains.

## Appendix 1

Three options exist for adjusting the cost of interventions to their 2009 USD equivalent.

**Method 1: **Costs of interventions are first converted from local currency to US dollars using the exchange rate at the year of costing and then inflated to 2009 USD using USD inflation rates.

**Method 2: **Costs are first inflated to 2009 values in the local currency and then converted to 2009 USD using 2009 exchange rates.

**Method 3: **For studies where an ingredients approach to costing has been used, a more detailed estimate of the inflated cost can be obtained. The cost of an intervention can be split into tradable costs (e.g. nets, insecticide, drugs, treatment kits) and non-tradable costs (e.g. personnel, training, information, education and communication (IEC)). Tradable costs are first converted into US dollars using exchange rates at the year of costing and then inflated to 2009 USD. Non-tradable costs are first inflated to 2009 values in the local currency and then converted to 2009 USD using 2009 conversion rates.

There are strengths and weaknesses associated with all the methods [27]. In this study, costs are inflated using method 1 (converting then inflating) as the costs published in many studies had already been converted to US dollars and many studies did not use an ingredients approach so it was not possible to identify the tradable and non-tradable cost components.

The three methods for inflating the costs of interventions are compared with the following examples:

**Ngugi *et al ***[56](Table 1)**: **In a study in coastal and western Kenya Ngugi *et al *[56] evaluated the cost to employers of distributing ITNs to employees to be $15.80 per net delivered in 2002 prices. 48% of the costs were classified as tradable and 52% as non-tradable. The exchange rates were 1 USD = 78 KSH in 2002 and 1 USD = 82 KSH in 2009.

**Table 1:** Methods for inflating the financial cost of ITNs as reported by Ngugi *et al *[56] in 2002 USD to 2009 USD.

		2002		2009	
		KSH	USD	KSH	USD
**Method 1**		-	15.80	-	19.20

**Method 2**		1232.40	15.80	1497.86	18.27

**Method 3**	tradable	591.24	7.58	-	9.22
	non-tradable	640.85	8.22	778.89	9.50
					
			15.80		18.72

Using method 1 (converting then inflating) the cost per net distributed was estimated to be 2009 USD 19.20, and using method 2 (inflating then converting) the cost was estimated to be 2009 USD 18.27. Using method 3 resulted in the intermediate estimate of 2009 USD 18.72. As the Kenyan Shilling was stable against the dollar there is not much difference between the three methods.

**Hanson *et al ***[57](Table 2)**: **In a Tanzanian study Hanson *et al *[57] evaluated the cost of distributing ITNs through a social marketing campaign. The cost per net delivered was estimated to be $11.90 in 2000 prices. 49.6% of the costs were classified as tradable and 50.4% as non-tradable. The exchange rates were 1 USD = 803 TSH in 2000 and 1 USD = 1355 TSH in 2009.

**Table 2:** Methods for inflating the financial cost of ITNs as reported by Hanson *et al *[57] in 2002 USD to 2009 USD.

		2002		2009	
		TSH	USD	TSH	USD
**Method 1**		-	11.90	-	15.37

**Method 2**		9556	11.90	12345	9.11

**Method 3**	tradable	4736	5.90	-	7.63
	non-tradable	4816	6.00	6222	4.59
					
			11.90		12.22

Using method 1 (converting then inflating) the cost per net distributed was estimated to be 2009 USD 15.37, and using method 2 (inflating then converting) the cost was estimated to be 2009 USD 9.11. Using method 3 resulted in the intermediate estimate of 2009 USD 12.22. As the Tanzanian Shilling experienced high inflation relative to the dollar there is significant variation between the estimates of cost.

## Competing interests

The authors declare that they have no competing interests.

## Authors' contributions

MTW, LC, RC and ACG outlined the scope of the review. MTW performed the review. MTW wrote the first draft of the manuscript. All authors contributed to the final version of the manuscript.

## Supplementary Material

Additional file 1 **CostReview**. Excel spreadsheet containing detailed information costing studies reviewed. Click here for file

Additional file 2 **Table S1**. Table of financial, standardized financial and economic cost of distributing and/or re-treating insecticide treated nets. Click here for file

Additional file 3 **Table S2**. Table of financial and economic costs per person protected by indoor residual spraying. Click here for file

Additional file 4 **Table S3**. Table of financial and economic cost per course/dose of intermittent preventive treatment. Click here for file

Additional file 5 **Table S4**. Table of financial cost of malaria diagnosis and treatment. Click here for file

Additional file 6 **Table S5**. Table of financial and economic costs of treating an episode of uncomplicated/severe malaria at health centres or hospitals (inpatient or outpatient). Click here for file

Additional file 7 **Table S6**. Table of cost-effectiveness of malaria control interventions. Click here for file
