# Supplementary material for: Costs and cost-effectiveness of malaria control interventions - a systematic review
Source: Malar J. 2011 Nov 3;10:337. doi: 10.1186/1475-2875-10-337 (PMC3229472; doi:10.1186/1475-2875-10-337)
Supplement: Additional file 2 — Table S1. Table of financial, standardized financial and economic cost of distributing and/or re-treating insecticide treated nets. [file 1475-2875-10-337-S2.DOC]

Table S1: Financial, standardized financial and economic cost of distributing and/or re-treating insecticide treated nets. Studies are broken down by region: Asia, South America and Africa. All studies were undertaken from a provider perspective, except those marked with * which were undertaken from a societal perspective. All costs are in 2009 USD.

| **Country** | **Description** | **Costing year** | **Financial Cost** | **Economic Cost** | **Annual financial cost** | **Reference** |
| --- | --- | --- | --- | --- | --- | --- |
| Thailand | Thailand campaign – treatment of existing ITN | 1994 | 2.97 | - | 0.99 | Kamolratanakul |
| India | India campaign – retreatment | 1997 | 3.33 | - | 1.11 | Bhatia |
| Colombia | Nearby treatment campaign – treatment only | 2001 | 6.37 | - | 2.12 | Kroeger |
| Colombia | Far away treatment campaign – treatment only | 2001 | 15.49 | - | 5.16 | Kroeger |
| Kenya | Campaign – children – free nets | 1996 | - | 3.11 | - | Wiseman |
| South Africa | Community campaign – free nets | 1999 | 13.35 | 10.05 | 5.76 | Goodman |
| Malawi | Government – social marketing – subsidized nets | 1999 | 5.70 | 3.47 | 9.54 | Stevens |
| Tanzania | Social marketing – subsidized nets | 2000 | 15.37 | 2.97 | 8.66 | Hanson |
| Kenya | Campaign – highly subsidized nets | 2000 | 4.42 | - | 0.88 | Guyatt |
| Kenya | ANC delivery – district level – free nets | 2001 | 4.76 | - | 1.59 | Guyatt |
| Kenya | ANC delivery – national level – free nets | 2001 | 5.00 | - | 1.67 | Guyatt |
| Ghana | Measles vaccine delivery – outreach campaign – free nets | 2002 | 4.50 | - | 1.50 | Grabowsky |
| Kenya | Employer campaign – subsidized nets | 2002 | 19.20 | - | 6.40 | Ngugi |
| Togo | Measles vaccine delivery – outreach campaign – free nets – LLIN | 2004 | 6.96 | 5.16 | 2.32 | Mueller |
| Senegal | Government – vouchers to pregnant women and children – LLIN | 2005 | 11.77 | 9.17 | 7.97 | Yukich |
| Eritrea | National government campaign – free nets | 2005 | 5.37 | 4.53 | 1.63 | Yukich |
| Tanzania | National campaign – ANC – pregnant women and U5 | 2005 | 6.68 | 5.47 | 3.02 | Yukich |
| DR Congo | ANC delivery – free nets – LLIN | 2005 | 10.84 | - | 3.61 | Becker-Dreps |
| Tanzania | Voucher scheme – subsidized nets – LLIN | 2006 | 8.47 | 7.46 | 2.82 | Mulligan |
| Burkina Faso | ANC delivery – free nets – LLIN | 2006 | 7.94 | - | 1.59 | De Allegri |
| Burkina Faso | Social marketing – subsidized nets – LLINs | 2006 | 8.90 | - | 1.78 | De Allegri |
| Zanzibar | ANC delivery – free nets – LLIN | 2006 | 8.75 | 4.12 | 2.19 | WHO |
| Uganda | Jinja campaign – pregnant women and children – free nets – LLIN | 2007 | 6.61 | 3.07 | 2.20 | Kolaczinski |
| Uganda | Adjumani campaign – pregnant women and children– free nets – LLIN | 2007 | 7.55 | 3.79 | 2.52 | Kolaczinski |
| Uganda | Adjumani – ANC delivery– free nets – LLIN | 2007 | 7.03 | 4.68 | 2.34 | Kolaczinski |
| Uganda | Campaign – free GF nets to pregnant women and children – LLIN | 2007 | 7.31 | 3.31 | 1.83 | WHO |
| Kenya | ANC sales 2007 – subsidized nets – LLIN | 2007 | 8.90 | 5.00 | 2.97 | WHO |
| Kenya | Rural sales 2007 – subsidized nets – LLIN | 2007 | 6.01 | 4.14 | 2.00 | WHO |
| Kenya | Urban sales 2007 – subsidized nets – LLIN | 2007 | 7.16 | 5.36 | 2.39 | WHO |
| Zanzibar | Campaign – children – free nets – LLIN | 2008 | 8.03 | 3.65 | 2.01 | WHO |
| Kenya | ANC sales 2008 – subsidized nets – LLIN | 2008 | 7.02 | 4.07 | 2.34 | WHO |
| Kenya | Rural sales 2008 – subsidized nets – LLIN | 2008 | 6.96 | 4.16 | 2.32 | WHO |
| Uganda | Campaign – free UNICEF pregnant women and children – LLIN | 2009 | 6.87 | 3.23 | 1.72 | WHO |
| Zanzibar | Campaign – everyone – free nets – LLIN | 2009 | 7.76 | 3.54 | 1.94 | WHO |
| Kenya | Campaign – highly subsidized nets* | 2000 | 5.44 | 3.02 | 1.09 | Guyatt |
| Tanzania | Voucher scheme – subsidized nets – LLIN* | 2006 | 9.35 | 8.34 | 3.12 | Mulligan |
| Burkina Faso | ANC delivery – free nets – LLIN* | 2006 | - | 5.30 | - | De Allegri |
| Burkina Faso | Social marketing – subsidized nets – LLINs* | 2006 | - | 5.30 | - | De Allegri |

**References**

1. Kamolratanakul P, Butraporn P, Prasittisuk M, Prasittisuk C, Indaratna K: **Cost-effectiveness and sustainability of lambdacyhalothrin-treated mosquito nets in comparison to DDT spraying for malaria control in western Thailand**. *American Journal of Tropical Medicine and Hygiene* 2001, **65**(4):279-284.

2. Bhatia MR, Fox-Rushby J, Mills A: **Cost-effectiveness of malaria control interventions when malaria mortality is low: insecticide-treated nets versus in-house residual spraying in India**. *Social Science & Medicine* 2004, **59**(3):525-539.

3. Kroeger A, Ayala C, Lara AM: **Unit costs for house spraying and bednet impregnation with residual insecticides in Colombia: a management tool for the control of vector-borne disease**. *Annals of Tropical Medicine and Parasitology* 2002, **96**(4):405-416.

4. Wiseman V, Hawley WA, ter Kuile FO, Phillips-Howard PA, Vulule JM, Nahlen BL, Mills AJ: **The cost-effectiveness of permethrin-treated bed nets in an area of intense malaria transmission in western Kenya**. *American Journal of Tropical Medicine and Hygiene* 2003, **68**(4):161-167.

5. Goodman CA, Mnzava AEP, Dlamini SS, Sharp BL, Mthembu DJ, Gumede JK: **Comparison of the cost and cost-effectiveness of insecticide-treated bednets and residual house-spraying in KwaZulu-Natal, South Africa**. *Tropical Medicine & International Health* 2001, **6**(4):280-295.

6. Stevens W, Wiseman V, Ortiz J, Chavasse D: **The costs and effects of a nationwide insecticide-treated net programme: the case of Malawi**. *Malaria Journal* 2005, **4**.

7. Hanson K, Kikumbih N, Schellenberg JA, Mponda H, Nathan R, Lake S, Mills A, Tanner M, Lengeler C: **Cost-effectiveness of social marketing of insecticide-treated nets for malaria control in the United Republic of Tanzania**. *Bulletin of the World Health Organization* 2003, **81**(4):269-276.

8. Guyatt HL, Kinnear J, Burini M, Snow RW: **A comparative cost analysis of insecticide-treated nets and indoor residual spraying in highland Kenya**. *Health Policy and Planning* 2002, **17**(2):144-153.

9. Guyatt HL, Corlett SK, Robinson TP, Ochola SA, Snow RW: **Malaria prevention in highland Kenya: indoor residual house-spraying vs. insecticide-treated bednets**. *Tropical Medicine & International Health* 2002, **7**(4):298-303.

10. Grabowsky M, Nobiya T, Ahun M, Donna R, Lengor M, Zimmerman D, Ladd H, Hoekstra E, Bello A, Baffoe-Wilmot A *et al*: **Distributing insecticide-treated bednets during measles vaccination: a low-cost means of achieving high and equitable coverage**. *Bulletin of the World Health Organization* 2005, **83**(3):195-201.

11. Ngugi IK, Chiguzo AN, Guyatt HL: **A cost analysis of the employer-based bednet programme in Coastal and Western Kenya**. *Health Policy and Planning* 2004, **19**(2):111-119.

12. Mueller DH, Wiseman V, Bakusa D, Morgah K, Dare A, Tchamdja P: **Cost-effectiveness analysis of insecticide-treated net distribution as part of the Togo Integrated Child Health Campaign**. *Malaria Journal* 2008, **7**.

13. Yukich JO, Tediosi F, Lengeler C: **Operations, costs and cost-effectiveness of five insecticide-treated net programs (Eritrea, Malawi, Tanzania, Togo, Senegal) and two indoor residual spray programs (Kwa-Zulu-Natal, Mozambique)**. 2007.

14. Yukich JO, Zerom M, Ghebremeskel T, Tediosi F, Lengeler C: **Costs and cost-effectiveness of vector control in Eritrea using insecticide-treated bed nets**. *Malaria Journal* 2009, **8**.

15. Yukich JO, Lengeler C, Tediosi F, Brown N, Mulligan J-A, Chavasse D, Stevens W, Justino J, Conteh L, Maharaj R *et al*: **Costs and consequences of large-scale vector control for malaria**. *Malaria Journal* 2008, **7**.

16. Becker-Dreps SI, Biddle AK, Pettifor A, Musuamba G, Imbie DN, Meshnick S, Behets F: **Cost-effectiveness of adding bed net distribution for malaria prevention to antenatal services in Kinshasa, Democratic Republic of the Congo**. *American Journal of Tropical Medicine and Hygiene* 2009, **81**(3):496-502.

17. Mulligan JA, Yukich J, Hanson K: **Costs and effects of the Tanzanian national voucher scheme for insecticide-treated nets**. *Malaria Journal* 2008, **7**.

18. De Allegri M, Marschall P, Flessa S, Tiendrebeogo J, Kouyate B, Jahn A, Muller O: **Comparative cost analysis of insecticide-treated net delivery strategies: sales supported by social marketing and free distribution through antenatal care**. *Health Policy and Planning*, **25**(1):28-38.

19. WHO: **Costing of ZMCP ITN Program - Report of a Consultation**. 2009.

20. Kolaczinski JH, Kolaczinski K, Kyabayinze D, Strachan D, Temperley M, Wijayanandana N, Kilian A: **Costs and effects of two public sector delivery channels for long-lasting insecticidal nets in Uganda**. *Malaria Journal*, **9**.

21. WHO: **Costing of Uganda ITN activities - Report of a Consultation**. 2009.

22. WHO: **Costing of PSI-Kenya ITN Program - Report of a Consultation**. 2009.
