# Supplementary material for: Costs and cost-effectiveness of malaria control interventions - a systematic review
Source: Malar J. 2011 Nov 3;10:337. doi: 10.1186/1475-2875-10-337 (PMC3229472; doi:10.1186/1475-2875-10-337)
Supplement: Additional file 3 — Table S2. Table of financial and economic costs per person protected by indoor residual spraying. [file 1475-2875-10-337-S3.DOC]

Table S1: Financial and economic costs per person protected by indoor residual spraying. All studies used a provider perspective. Studies are broken down by region: Asia, South America and Africa. Costs are in 2009 USD.

| **Country** | **Delivery** | **Insecticide & frequency** | **Costing year** | **Financial cost** | **Economic cost** | **Annual financial cost** | **Reference** |
| --- | --- | --- | --- | --- | --- | --- | --- |
| Thailand |  | DDT | 1994 | 3.91 | - | 7.82 | Kamolratanakul |
| India |  | deltamethrin | 1997 | 1.94 | - | 3.88 | Bhatia |
| Colombia | houses near spray centre | lambdacyhalothrin twice yearly | 2001 | 9.25 | - | 9.25 | Kroeger |
| Colombia | houses far from spray centre | lambdacyhalothrin twice yearly | 2001 | 12.87 | - | 12.87 | Kroeger |
| South Africa |  | pyrethroid, yearly | 1999 | 3.35 | 3.15 | 6.70 | Goodman |
| Zimbabwe |  | deltamethrin, single round | 2000 | 1.54 | - | 3.08 | Worrall |
| Kenya |  | lambdacyhalothrin, single round | 2000 | 1.11 | 1.14 | 2.22 | Guyatt |
| Mozambique | rural, continuous campaign | Ficam, two rounds per year | 2001 | 6.78 | 6.23 | 6.78 | Conteh |
| Mozambique | peri-urban, continuous campaign | Ficam, two rounds per year | 2001 | 4.04 | 3.66 | 4.04 | Conteh |

**References**

1. Kamolratanakul P, Butraporn P, Prasittisuk M, Prasittisuk C, Indaratna K: **Cost-effectiveness and sustainability of lambdacyhalothrin-treated mosquito nets in comparison to DDT spraying for malaria control in western Thailand**. *American Journal of Tropical Medicine and Hygiene* 2001, **65**(4):279-284.

2. Bhatia MR, Fox-Rushby J, Mills A: **Cost-effectiveness of malaria control interventions when malaria mortality is low: insecticide-treated nets versus in-house residual spraying in India**. *Social Science & Medicine* 2004, **59**(3):525-539.

3. Kroeger A, Ayala C, Lara AM: **Unit costs for house spraying and bednet impregnation with residual insecticides in Colombia: a management tool for the control of vector-borne disease**. *Annals of Tropical Medicine and Parasitology* 2002, **96**(4):405-416.

4. Goodman CA, Mnzava AEP, Dlamini SS, Sharp BL, Mthembu DJ, Gumede JK: **Comparison of the cost and cost-effectiveness of insecticide-treated bednets and residual house-spraying in KwaZulu-Natal, South Africa**. *Tropical Medicine & International Health* 2001, **6**(4):280-295.

5. Worrall E, Connor SJ, Thomson MC: **Improving the cost-effectiveness of IRS with climate informed health surveillance systems**. *Malaria Journal* 2008, **7**.

6. Guyatt HL, Kinnear J, Burini M, Snow RW: **A comparative cost analysis of insecticide-treated nets and indoor residual spraying in highland Kenya**. *Health Policy and Planning* 2002, **17**(2):144-153.

7. Conteh L, Sharp BL, Streat E, Barreto A, Konar S: **The cost and cost-effectiveness of malaria vector control by residual insecticide house-spraying in southern Mozambique: a rural and urban analysis**. *Tropical Medicine & International Health* 2004, **9**(1):125-132.
