# Supplementary material for: Costs and cost-effectiveness of malaria control interventions - a systematic review
Source: Malar J. 2011 Nov 3;10:337. doi: 10.1186/1475-2875-10-337 (PMC3229472; doi:10.1186/1475-2875-10-337)
Supplement: Additional file 4 — Table S3. Table of financial and economic cost per course/dose of intermittent preventive treatment. [file 1475-2875-10-337-S4.DOC]

Table S1: Financial and economic cost per course/dose of intermittent preventive treatment. Studies are grouped according to whether IPT was administered to pregnant women (p), children (c) or infants (i). The number of courses administered is indicated in the description column. All costs are from a provider perspective. All costs are in 2009 USD.

| **Country** | **Description** | **Costing year** | **Financial cost** | **Economic cost** | **Annual financial cost** | **Reference** |
| --- | --- | --- | --- | --- | --- | --- |
| SSA – low income | IPTp-CQ ANC (2 doses) | 1995 | 1.89 | - | 1.89 | Goodman |
| SSA – low income | IPTp-SP ANC (2 doses) | 1995 | 1.64 | - | 1.64 | Goodman |
| SSA – middle income | IPTp-CQ ANC (2 doses) | 1995 | 2.06 | - | 2.06 | Goodman |
| SSA – middle income | IPTp-SP ANC (2 doses) | 1995 | 1.82 | - | 1.82 | Goodman |
| SSA – middle income | IPTp-CQ ANC (2 doses) | 1995 | 3.36 | - | 3.36 | Goodman |
| SSA – middle income | IPTp-SP ANC (2 doses) | 1995 | 3.11 | - | 3.11 | Goodman |
| Uganda | IPTp-SP community (2 doses) | 2003 | 2.43 | - | 2.43 | Mbonye |
| Uganda | IPTp-SP health centre (2 doses) | 2003 | 2.66 | - | 2.66 | Mbonye |
| Mozambique | IPTp-SP ANC (2 doses) | 2007 | 0.47 | - | 0.47 | Sicuri |
| Kenya | IPTc-SP schools (1 year = 3 doses) | 2006 | 1.25 | 1.95 | 1.25 | Temperley |
| Ghana | IPTc-SP bimonthly CHW (6 months = 3 doses) (trial) | 2008 | 4.96 | 8.22 | 9.92 | Conteh |
| Ghana | IPTc-AQ&AS monthly CHW (6 months = 6 doses) (trial) | 2008 | 11.80 | 14.85 | 23.60 | Conteh |
| Ghana | IPTc-AQ&AS bimonthly CHW (6 months = 3 doses) (trial) | 2008 | 7.35 | 10.71 | 14.70 | Conteh |
| Ghana | IPTc-SP bimonthly district (6 months = 3 doses) (district) | 2008 | - | 1.87 | - | Conteh |
| Ghana | IPTc-AQ&AS monthly district (6 months = 6 doses) (district) | 2008 | - | 4.35 | - | Conteh |
| Ghana | IPTc-AQ&AS bimonthly district (6 months = 3 doses) (district) | 2008 | - | 2.70 | - | Conteh |
| Ghana | IPTc-SP&AQ VHW community (4 courses) | 2008 | 3.24 | 4.60 | 3.24 | Patouillard |
| Ghana | IPTc-SP&AQ VHW facility (4 courses) | 2008 | 3.84 | 5.29 | 3.84 | Patouillard |
| Ghana | IPTc-SP&AQ outpatient dept (4 courses) | 2008 | 3.52 | 4.95 | 3.52 | Patouillard |
| Ghana | IPTc-SP&AQ EPI (4 courses) | 2008 | 4.22 | 5.67 | 4.22 | Patouillard |
| Tanzania | IPTi-SP with EPI (1 dose) | 2005 | 0.18 | 0.23 | 1.08 | Manzi |
| Tanzania | IPTi-SP with EPI (1 dose) | 2006 | 0.08 | 0.13 | 0.48 | Hutton |
| Mozambique | IPTi-SP with EPI (1 dose) | 2006 | 0.10 | 0.15 | 0.60 | Hutton |

**References**

1. Goodman CA, Coleman PG, Mills AJ: **The cost-effectiveness of antenatal malaria prevention in sub-Saharan Africa**. *American Journal of Tropical Medicine and Hygiene* 2001, **64**(1-2):45-56.

2. Mbonye AK, Hansen KS, Bygbjerg IC, Magnussen P: **Intermittent preventive treatment of malaria in pregnancy: the incremental cost-effectiveness of a new delivery system in Uganda**. *Transactions of the Royal Society of Tropical Medicine and Hygiene* 2008, **102**(7):685-693.

3. Sicuri E, Bardaji A, Nhampossa T, Maixenchs M, Nhacolo A, Nhalungo D, Alonso PL, Menendez C: **Cost-Effectiveness of Intermittent Preventive Treatment of Malaria in Pregnancy in Southern Mozambique**. *Plos One*, **5**(10).

4. Temperley M, Mueller DH, Njagi JK, Akhwale W, Clarke SE, Jukes MCH, Estambale BBA, Brooker S: **Costs and cost-effectiveness of delivering intermittent preventive treatment through schools in western Kenya**. *Malaria Journal* 2008, **7**.

5. Conteh L, Patouillard, E, Kweku, M, Legood, R, Greenwood, B, Chandramohan, D,: **Cost Effectiveness of Seasonal Intermittent Preventive Treatment using Amodiaquine & Artesunate or Sulphadoxine-Pyrimethamine in Ghanaian Children**. *PLoS One* 2010.

6. Patouillard E, Conteh L, Webster J, Kweku M, Chandramohan D, Greenwood B: **Coverage, adherence and costs of intermittent preventive treatment of malaria in children employing different delivery strategies in Jasikan district, Ghana (in press)**. *PLoS One* 2011.

7. Manzi F, Hutton G, Schellenberg J, Tanner M, Alonso P, Mshinda H, Schellenberg D: **From strategy development to routine implementation: the cost of Intermittent Preventive Treatment in Infants for malaria control**. *Bmc Health Services Research* 2008, **8**.

8. Hutton G, Schellenberg D, Tediosi F, Macete E, Kahigwa E, Sigauque B, Mas X, Trapero M, Tanner M, Trilla A *et al*: **Cost-effectiveness of malaria intermittent preventive treatment in infants (IPTi) in Mozambique and the United Republic of Tanzania**. *Bulletin of the World Health Organization* 2009, **87**(2):123-129.
