# Supplementary material for: Costs and cost-effectiveness of malaria control interventions - a systematic review
Source: Malar J. 2011 Nov 3;10:337. doi: 10.1186/1475-2875-10-337 (PMC3229472; doi:10.1186/1475-2875-10-337)
Supplement: Additional file 5 — Table S4. Table of financial cost of malaria diagnosis and treatment. [file 1475-2875-10-337-S5.DOC]

Table S1: Financial cost of malaria diagnosis and treatment. The financial cost per person diagnosed and, where available, the cost per person diagnosed and treated successfully are presented. Studies were undertaken from a provider perspective, except those marked with * which were undertaken from a societal perspective. All costs are in 2009 USD.

| **Country** | **Description** | **Costing year** | **Financial cost of diagnosis** | **Financial cost of diagnosis and treatment** | **Reference** |
| --- | --- | --- | --- | --- | --- |
| Sri Lanka | Microscopy – health facility | 2002 | 0.34 | - | Fernando |
| Sri Lanka | RDT: immunochromato-graphic test for *P. vivax* – health facility | 2002 | 4.64 | - | Fernando |
| Thailand | microscopy and ACT –mobile clinic, children | 2000 | - | 10.05 | Bualombai |
| Thailand | RDT-Optimal and ACT –mobile clinic, children | 2000 | - | 8.23 | Bualombai |
| Thailand | RDT-ICT and ACT – mobile clinic, children | 2000 | - | 8.96 | Bualombai |
| Thailand | microscopy and ACT – mobile clinic*, children | 2000 | - | 13.31 | Bualombai |
| Thailand | RDT-Optimal and ACT – mobile clinic*, children | 2000 | - | 8.34 | Bualombai |
| Thailand | RDT-ICT and ACT – mobile clinic*, children | 2000 | - | 9.20 | Bualombai |
| Brazil | Microscopy – health facility (model) | 2006 | 7.48 | - | de Oliveira |
| Brazil | RDT-Optimal – health facility (model) | 2006 | 5.66 | - | de Oliveira |
| Zambia | clinical diagnosis – clinic and ACT | 2005 | 3.07 | 4.78 | Chanda |
| Zambia | microscopy – clinic and ACT | 2005 | 9.34 | 10.70 | Chanda |
| Zambia | RDT – clinic and ACT | 2005 | 5.35 | 6.72 | Chanda |
| Sub-Saharan Africa | CHW presumptive AS&AQ (PfPR=0.25) – CHW (model) | 2006 | - | 14.06 | Rolland |
| Sub-Saharan Africa | CHW RDT AS&AQ (PfPR=0.25) – CHW (model) | 2006 | - | 15.19 | Rolland |
| Sub-Saharan Africa | CHW presumptive AS&AQ (PfPR=0.5) – CHW (model) | 2006 | - | 7.03 | Rolland |
| Sub-Saharan Africa | CHW RDT AS&AQ (PfPR=0.5) – CHW (model) | 2006 | - | 8.44 | Rolland |
| Sub-Saharan Africa | CHW presumptive AS&AQ (PfPR=0.75) | 2006 | - | 4.64 | Rolland |
| Sub-Saharan Africa | CHW RDT AS&AQ (PfPR=0.75) – CHW (model) | 2006 | - | 6.19 | Rolland |
| Ethiopia | Presumptive treatment with AL – health post | 2007 | - | 11.08 | Lemma |
| Ethiopia | Paracheck: RDT – AL for *Pf*, CQ otherwise – health post | 2007 | - | 4.66 | Lemma |
| Ethiopia | Parascreen: RDT – AL for *Pf*, referral otherwise – health post | 2007 | - | 1.69 | Lemma |
| Tanzania | Microscopy with ACT – health facility | 2008 | - | 2.32 | Yukich |
| Tanzania | RDT with ACT – health facility | 2008 | - | 3.63 | Yukich |
| Tanzania | Microscopy with ACT – health facility* | 2008 | - | 3.68 | Yukich |
| Tanzania | RDT with ACT – health facility* | 2008 | - | 4.72 | Yukich |
| Nigeria | Clinical diagnosis of children and ART & AQ – hospital* | 2009 | - | 4.49 | Parikh |
| Nigeria | Clinical diagnosis of adults and ART & SP – hospital* | 2009 | - | 4.14 | Parikh |
| Nigeria | Clinical diagnosis of adults and ART & AQ – hospital* | 2009 | - | 4.63 | Parikh |
| Nigeria | Microscopy diagnosis of children and ART & AQ – hospital* | 2009 | 3.74 | 6.04 | Parikh |
| Nigeria | Microscopy diagnosis of adults and ART & SP – hospital* | 2009 | 3.74 | 4.84 | Parikh |
| Nigeria | Microscopy diagnosis of adults and ART & AQ – hospital* | 2009 | 3.74 | 4.97 | Parikh |
| Zambia | RDT diagnosis by home management, ACT treatment | 2009 | 3.99 | 4.22 | Chanda |
| Zambia | Diagnosis at health facility, ACT treatment | 2009 | 5.33 | 6.61 | Chanda |

**References**

1. Fernando SD, Karunaweera ND, Fernando WP, Attanayake N, Wickremasinghe AR: **A cost analysis of the use of the rapid, whole-blood, immunochromatographic P.f/P.v assay for the diagnosis of Plasmodium vivax malaria in a rural area of Sri Lanka**. *Annals of Tropical Medicine and Parasitology* 2004, **98**(1):5-13.

2. Bualombai P, Prajakwong S, Aussawatheerakul N, Congpoung K, Sudathip S, Thimasarn K, Sirichaisinthop J, Indaratna K, Kidson C, Srisuphanand M: **Determining cost-effectiveness and cost component of three malaria diagnostic models being used in remote non-microscope areas**. *Southeast Asian Journal of Tropical Medicine and Public Health* 2003, **34**(2):322-333.

3. de Oliveira MRF, Gomes AD, Toscano CM: **Cost effectiveness of OptiMal (R) rapid diagnostic test for malaria in remote areas of the Amazon Region, Brazil**. *Malaria Journal*, **9**.

4. Chanda P, Castillo-Riquelme M, Masiye F: **Cost-effectiveness analysis of the available strategies for diagnosing malaria in outpatient clinics in Zambia**. *Cost Effectiveness and Resource Allocation* 2009, **7**(5):(8 April 2009).

5. Rolland E, Checchi F, Pinoges L, Balkan S, Guthmann JP, Guerin PJ: **Operational response to malaria epidemics: are rapid diagnostic tests cost-effective?** *Tropical Medicine & International Health* 2006, **11**(4):398-408.

6. Lemma H, San Sebastian M, Lofgren C, Barnabas G: **Cost-effectiveness of three malaria treatment strategies in rural Tigray, Ethiopia where both Plasmodium falciparum and Plasmodium vivax co-dominate**. *Cost effectiveness and resource allocation : C/E* 2011, **9**:2.

7. Yukich J: **Lack of cost-savings from Rapid Diagnostics Tests for malaria in Dar es Salaam, Tanzania**. *PhD Thesis, Swiss Tropical Institute* 2009.

8. Parikh R, Amole I, Tarpley M, Gbadero D, Davidson M, Vermund SH: **Cost comparison of microscopy vs. empiric treatment for malaria in southwestern nigeria: a prospective study**. *Malaria Journal* 2010, **9**.

9. Chanda P, Hamainza B, Moonga HB, Chalwe V, Banda P, Pagnoni F: **Relative costs and effectiveness of treating uncomplicated malaria in two rural districts in Zambia: implications for nationwide scale-up of home-based management**. *Malaria Journal* 2011, **10**.
