# Supplementary material for: Costs and cost-effectiveness of malaria control interventions - a systematic review
Source: Malar J. 2011 Nov 3;10:337. doi: 10.1186/1475-2875-10-337 (PMC3229472; doi:10.1186/1475-2875-10-337)
Supplement: Additional file 6 — Table S5. Table of financial and economic costs of treating an episode of uncomplicated/severe malaria at health centres or hospitals (inpatient or outpatient). [file 1475-2875-10-337-S6.DOC]

Table S1: Financial and economic costs of treating an episode of uncomplicated/severe malaria at health centres or hospitals (inpatient or outpatient). The studies are broken down by region (Asia and Africa) and then by treatment type: treatment of severe malaria as hospital inpatients or treatment of uncomplicated malaria as outpatients or at health facilities. Studies were undertaken from a provider perspective, except those marked with * which were undertaken from a societal perspective. All costs are in 2009 USD. ^+^This study used a model to evaluate the cost to the health system incorporating both outpatients and inpatients.

| **Country** | **Description** | **Costing year** | **Financial cost** | **Economic cost** | **Reference** |
| --- | --- | --- | --- | --- | --- |
| India | hospital treatment CQ – hospital inpatients | 1998 | 15.64 | - | Gogtay |
| India | hospital treatment MQ – hospital inpatients | 1998 | 18.86 | - | Gogtay |
| India | hospital treatment CQ – hospital inpatients* | 1998 | 28.65 | - | Gogtay |
| India | hospital treatment MQ – hospital inpatients* | 1998 | 31.87 | - | Gogtay |
| Bangladesh | severe treatment AS – hospital inpatients, children | 2008 | - | 45.67 | Lubell |
| India | severe treatment AS – hospital inpatients, children | 2008 | - | 72.97 | Lubell |
| Indonesia | severe treatment AS – hospital inpatient, children | 2008 | - | 69.55 | Lubell |
| Myanmar | severe treatment AS – hospital inpatients, children | 2008 | - | 27.92 | Lubell |
| Bangladesh | severe treatment quinine – hospital inpatients, children | 2008 | - | 36.33 | Lubell |
| India | severe treatment quinine – hospital inpatients, children | 2008 | - | 62.70 | Lubell |
| Indonesia | severe treatment quinine – hospital inpatients, children | 2008 | - | 66.22 | Lubell |
| Myanmar | severe treatment quinine – hospital inpatients, children | 2008 | - | 26.99 | Lubell |
| Papua New Guinea | Clinic treatment of uncomplicated *P. falciparum* with CQ & SP*, children | 2008 | 3.63 | - | Davis |
| Papua New Guinea | Clinic treatment of uncomplicated *P. falciparum* with ART & SP*, children | 2008 | 4.03 | - | Davis |
| Papua New Guinea | Clinic treatment of uncomplicated *P. falciparum* with DHA & PQ*, children | 2008 | 3.83 | - | Davis |
| Papua New Guinea | Clinic treatment of uncomplicated *P. falciparum* with AL*, children | 2008 | 4.63 | - | Davis |
| Papua New Guinea | Clinic treatment of uncomplicated *P. vivax* with CQ & SP* , children | 2008 | 4.18 | - | Davis |
| Papua New Guinea | Clinic treatment of uncomplicated *P. vivax* with ART & SP*, children | 2008 | 4.77 | - | Davis |
| Papua New Guinea | Clinic treatment of uncomplicated *P. vivax* with DHA & PQ*, children | 2008 | 4.08 | - | Davis |
| Papua New Guinea | Clinic treatment of uncomplicated *P.vivax* with AL*, children | 2008 | 5.39 | - | Davis |
| South Africa | health centre treatment CQ – health system^+^ | 1997 | 137.87 | - | Wilkins |
| South Africa | health centre treatment SP – health system^+^ | 1997 | 28.54 | - | Wilkins |
| South Africa | severe inpatient treatment SP– hospital inpatient | 2002 | - | 210.88 | Muheki |
| South Africa | severe inpatient treatment AL– hospital inpatient | 2002 | - | 288.79 | Muheki |
| Kenya | national hospital severe treatment – hospital inpatient, children | 2005 | 108.83 | - | Ayieko |
| Kenya | district hospital severe treatment – hospital inpatient, children | 2005 | 71.07 | - | Ayieko |
| Tanzania | quinine treatment for severe malaria - inpatient | 2009 | - | 65.50 | Lubell |
| Tanzania | artesunate treatment for severe malaria - inpatient | 2009 | - | 66.70 | Lubell |
| Tanzania | quinine treatment for severe malaria - inpatient | 2009 | - | 55.60 | Lubell |
| Tanzania | artesunate treatment for severe malaria - inpatient | 2009 | - | 54.00 | Lubell |
| Uganda | quinine treatment for severe malaria - inpatient | 2009 | - | 58.60 | Lubell |
| Uganda | artesunate treatment for severe malaria - inpatient | 2009 | - | 59.60 | Lubell |
| Nigeria | quinine treatment for severe malaria - inpatient | 2009 | - | 86.20 | Lubell |
| Nigeria | artesunate treatment for severe malaria - inpatient | 2009 | - | 108.70 | Lubell |
| sub-Saharan Africa | quinine treatment for severe malaria- inpatient (pooled analysis) | 2009 | - | 63.50 | Lubell |
| sub-Saharan Africa | artesunate treatment for severe malaria- inpatient (pooled analysis) | 2009 | - | 66.50 | Lubell |
| South Africa | health centre treatment SP– hospital outpatient | 2002 | - | 9.14 | Muheki |
| South Africa | health centre treatment AL – hospital outpatient | 2002 | - | 16.41 | Muheki |
| South Africa | outpatient treatment SP– hospital outpatient | 2002 | - | 28.55 | Muheki |
| South Africa | outpatient treatment AL– hospital outpatient | 2002 | - | 37.99 | Muheki |
| Tanzania | health centre treatment ACT – health centre | 2003 | 2.36 | - | Njau |
| Tanzania | children hospital treatment SP – hospital outpatient, children | 2005 | 5.80 | - | Wiseman |
| Tanzania | children hospital treatment AQ – hospital outpatient, children | 2005 | 5.84 | - | Wiseman |
| Tanzania | children hospital treatment AQ+SP – hospital outpatient, children | 2005 | 5.90 | - | Wiseman |
| Tanzania | children hospital treatment AQ+AS – hospital outpatient, children | 2005 | 6.33 | - | Wiseman |
| Tanzania | children hospital treatment AL – hospital outpatient, children | 2005 | 6.79 | - | Wiseman |
| Zambia | outpatient treatment with AL – health centre outpatient | 2005 | 8.36 | - | Chanda |
| Zambia | outpatient treatment with SP– health centre outpatient | 2005 | 7.05 | - | Chanda |
| Tanzania | children hospital treatment SP– hospital outpatient* | 2005 | 23.60 | - | Wiseman |
| Tanzania | children hospital treatment AQ– hospital outpatient* | 2005 | 23.65 | - | Wiseman |
| Tanzania | children hospital treatment AQ+SP– hospital outpatient* | 2005 | 12.88 | - | Wiseman |
| Tanzania | children hospital treatment AQ+AS– hospital outpatient* | 2005 | 11.56 | - | Wiseman |
| Tanzania | children hospital treatment AL– hospital outpatient* | 2005 | 11.15 | - | Wiseman |

**References**

1. Gogtay NJ, Kadam VS, Desai S, Kamtekar KD, Dalvi SS, Kshirsagar NA: **A cost-effectiveness analysis of three antimalarial treatments for acute, uncomplicated Plasmodium falciparum malaria in Mumbai, India**. *Journal of Association of Physicians of India* 2003, **51**:877-879.

2. Lubell Y, Yeung S, Dondorp AM, Day NP, Nosten F, Tjitra E, Faiz MA, Bin Yunus E, Anstey NM, Mishra SK *et al*: **Cost-effectiveness of artesunate for the treatment of severe malaria**. *Tropical Medicine & International Health* 2009, **14**(3):332-337.

3. Davis WA, Clarke PM, Siba PM, Karunajeewa HA, Davy C, Mueller I, Davis TME: **Cost-effectiveness of artemisinin combination therapy for uncomplicated malaria in children: data from Papua New Guinea**. *Bulletin of the World Health Organization* 2011, **89**(3):211-220.

4. Wilkins JJ, Folb PI, Valentine N, Barnes KI: **An economic comparison of chloroquine and sulfadoxine-pyrimethamine as first-line treatment for malaria in South Africa: development of a model for estimating recurrent direct costs**. *Transactions of the Royal Society of Tropical Medicine and Hygiene* 2002, **96**(1):85-90.

5. Muheki C, McIntyre D, Barnes KI: **Artemisinin-based combination therapy reduces expenditure on malaria treatment in KwaZulu Natal, South Africa**. *Tropical Medicine & International Health* 2004, **9**(9):959-966.

6. Ayieko P, Akumu AO, Griffiths UK, English M: **The economic burden of inpatient paediatric care in Kenya: household and provider costs for treatment of pneumonia, malaria and meningitis**. *Cost Eff Resour Alloc* 2009, **7**:3.

7. Lubell Y, Riewpaiboon A, Dondorp AM, von Seidlein L, Mokuolu OA, Nansumba M, Gesase S, Kent A, Mtove G, Olaosebikan R *et al*: **Cost-effectiveness of parenteral artesunate for treating children with severe malaria in sub-Saharan Africa**. *Bulletin of the World Health Organization* 2011, **89**(7):504-512.

8. Njau JD, Goodman CA, Kachur SP, Mulligan J, Munkondya JS, McHomvu N, Abdulla S, Bloland P, Mills A: **The costs of introducing artemisinin-based combination therapy: evidence from district-wide implementation in rural Tanzania**. *Malaria Journal* 2008, **7**.

9. Wiseman V, Kim M, Mutabingwa TK, Whitty CJM: **Cost-effectiveness study of three antimalarial drug combinations in Tanzania**. *Plos Medicine* 2006, **3**:1844-1850.

10. Chanda P, Masiye F, Chitah BM, Sipilanyambe N, Hawela M, Banda P, Okorosobo T: **A cost-effectiveness analysis of artemether lumefantrine for treatment of uncomplicated malaria in Zambia**. *Malaria Journal* 2007, **6**.
