# Supplementary material for: Costs and cost-effectiveness of malaria control interventions - a systematic review
Source: Malar J. 2011 Nov 3;10:337. doi: 10.1186/1475-2875-10-337 (PMC3229472; doi:10.1186/1475-2875-10-337)
Supplement: Additional file 7 — Table S6. Table of cost-effectiveness of malaria control interventions. [file 1475-2875-10-337-S7.DOC]

Table S6: Cost-effectiveness of malaria control interventions. All costs are in 2009 USD.

| **Reference** | **Study area and year** | **Intervention delivered and study group** | | **Perspective** | **Transmission intensity** | **Cost per death averted (US$)** | **Cost per DALY averted (US$)** | **Cost per case averted (US$)** | **Notes** |
| --- | --- | --- | --- | --- | --- | --- | --- | --- | --- |
| ***Prevention*** | |  | |  |  |  |  |  |  |
| **Insecticide Treated Nets** | | | | |  |  |  |  |  |
| Bhatia  2004 | Surat, India  1997 – 1998 | Whole population (deltamethrin nets) | | Provider |  | - | - | 71  (60, 85) | Intervention evaluated against early diagnosis and prompt treatment alone. |
|  |  |  | |  |  | - | - | 30 | Incremental cost-effectiveness of ITNs instead of IRS. |
|  |  |  | |  |  |  |  |  |  |
| Kamolratanakul 2001 | Thai-Myanmar border  1993 – 1994 | Whole villages – ITN (lambdacyhalothrin) | | Provider | incidence > 0.2 | - | - | 2.29 | This study costs the distribution of ITNs to those without nets and the re-treatment of existing nets in the population. |
|  |  |  | |  |  |  |  |  |  |
| BeckerDrepps 2009 | Kinshasha, DRC  2005 – 2006 | Young children and pregnant women – ANC | | Provider | *Pf*PR=0.6 | 468  (403, 1236) | 19.60  (9.72, 35) | - |  |
|  |  |  | |  |  |  |  |  |  |
| Yukich  2009 | Eritrea  2001 – 2005 | Whole population – mass distribution | | Provider |  | 1650  (1362, 6598) | 50  (25, 200) | - | These results are based on a study of the Eritrean National Malaria Control Programme. The deaths referred to are child deaths averted. The upper bound of the SA is for the case of a net providing only 3 months’ protection. |
|  |  | Whole population – re-treatment | |  |  | 499  (249, 1997) | 14.80  (7.97, 60) | - |  |
|  |  |  | |  |  |  |  |  |  |
| Mueller  2008 | Togo nation-wide  2004 | Children – nation-wide EPI | | Provider | incidence = 1.2 | 743 | 19.16  (3.56, 32) | 3.81  (0.71, 6.38) | From the Togo National Malaria Control Programmes. ITNs distributed alongside measles vaccines in the EPI. |
|  |  |  | |  |  |  |  |  |  |
| Yukich  2008 | Malawi nation- wide 1999-2005 | Children and pregnant women – ANC | | Provider |  | 1258 | 38 | - | Excluding ITN retreatment kits. Costs are economic costs. |
|  |  | Children and pregnant women – ANC + retreat | | Provider |  | 1391 | 42 | - | Including the cost of ITN retreatment. Costs are economic costs. |
|  |  |  | |  |  |  |  |  |  |
| Yukich  2008 | Senegal nation-wide 2000-2005 | Children and pregnant women – ANC | | Provider |  | 2504 | 76 | - | Excluding ITN retreatment kits. Costs are economic costs. |
|  |  | Children and pregnant women – ANC + retreat | | Provider |  | 3332 | 101 | - | Including the cost of ITN retreatment. Costs are economic costs. |
|  |  |  | |  |  |  |  |  |  |
| Yukich  2008 | Tanzania nation-wide 2002-2005 | Children and pregnant women – ANC | | Provider |  | 897 | 27 | - | Excluding ITN retreatment kits. Costs are economic costs. |
|  |  | Children and pregnant women – ANC + retreat | | Provider |  | 1987 | 60 | - | Including the cost of ITN retreatment. Costs are economic costs. |
|  |  |  | |  |  |  |  |  |  |
| Wiseman 2003 | Western Kenya  1996 – 1999 | Children 0-4 years (direct effect only) | | Society | Intense, perennial | 2311 | 92 | - | This study only presented a sensitivity analysis for community wide deaths averted and not the other endpoints. |
|  |  | Children 0-4 years (community effect) | |  |  | 1716  (1186, 1914) | 69 | - |  |
|  |  |  | |  |  |  |  |  |  |
|  |  |  | |  |  |  |  |  |  |
| Hanson  2003 | Rural Tanzania  1997 – 1999 | Children  0-4 years (social marketing) | | Societal | EIR = 200-300 | 2014  (758, 2014) | 73 (28, 73) | - | In this study, only factors that would make the intervention more cost-effective were considered in the sensitivity analysis. |
|  |  |  | |  |  |  |  |  |  |
| Guyatt  2002 | Kenyan highlands  1999-2000 | Whole population (deltamethrin nets) | | Provider | epidemic | - | - | 37 | This study was undertaken during an epidemic. Cases are infections averted. |
|  |  |  | |  |  |  |  |  |  |
| Goodman 2001 | KwaZulu Natal, SA  1998 – 1999 | Whole population – ITN vs. IRS | | Provider | Endemic, low | 2239  (819, 10243) | - | 21  (7.72, 97) | These estimates are for the cost-effectiveness of ITNs compared to a baseline of IRS. |
|  |  |  | |  |  |  |  |  |  |
| Coleman 1999 | Sub-Saharan Africa | Children  0-5 years – ITN | | Society | incidence = 1-2.9 | - | 62  (25, 120) | - | This study looks at possible rebound mortality after ITN distribution. Only the results with no rebound effects are included. |
|  |  |  | |  |  |  |  |  |  |
| Some  1999 | Kenya  1993 - 1994 | Children – ITN (delivery & treatment) | |  |  | 4298  (4124-4534) | - | - | This result is taken indirectly from a review by Goodman *et al*. Deaths are child deaths averted. |
|  |  |  | |  |  |  |  |  |  |
| Guyatt  1999 | Sub-Saharan Africa | Children  0-4 years – ITN (efficacy=0.18) | | Provider |  | - | 13.04 | - | The main aim of this study was to investigate the impact of ITNs on the acquisition of immunity, but the results are still of interest. ITN efficacy is against all cause mortality. Results presented here are cost per YLLs averted and not DALYs. |
|  |  | Children  0-4 years – ITN (efficacy=0.18) | |  |  | - | 8.15 | - |  |
|  |  | Children  0-9 years – ITN (efficacy=0.29) | |  |  | - | 22 | - |  |
|  |  | Children  0-9 years – ITN (efficacy=0.29) | |  |  | - | 13.62 | - |  |
|  |  |  | |  |  |  |  |  |  |
| Graves  1998 | The Gambia  1990 | Children 0-4 years – ITN (permethrin) | | Provider | incidence = 1 | 1235  (922, 1732) | - | 27  (20, 38) |  |
|  |  |  | |  |  |  |  |  |  |
| Aikins  1998 | The Gambia,  1992 – 1993 | Children  0-9 years – nationwide ITN campaign | | Society |  | 745  (566, 1215) | 50  (38, 81) | 43  (32, 70) | Gambian National Impregnated Bednet Programme. |
|  |  |  | |  |  |  |  |  |  |
| Binka  1997 | Northern Ghana | Children  0-9 years (delivery & treatment) | | Provider | EIR = 300 | 2986  (1403, 3237) | 110  (52, 119) | - |  |
|  |  |  | |  |  |  |  |  |  |
| Evans  1997 | West Africa (Gambian cost data) | Children  0-4 years – ITN distribution | | Provider | incidence = 0.8 | - | 15.35 – 31  (13.57, 186) | - | The upper bound of the sensitivity analysis corresponds to the case of 3 nets per child. |
|  |  |  | |  |  |  |  |  |  |
| WHO  1996 | Sub-Saharan Africa, 1990 | Children 0-4 years – ITN distribution | | Provider |  | - | 12.15 – 24.31 | - | Compliance between 50% and 100%. 25% reduction in all-cause child mortality in children < 5 years. |
|  |  |  | |  |  |  |  |  |  |
| Picard  1993 | The Gambia  1989 – 1990 | Children 0-4 years – ITN only | | Society |  | 326 | 13.72 | 49 |  |
|  |  | Children 0-4 years – ITN with chemoprophylaxis | |  |  | 447 | 18.83 | 34 |  |
| **Indoor Residual Spraying** | | | | |  |  |  |  |  |
| Bhatia  2004 | Surat, India  1997 – 1998 | Whole population – IRS | | Provider |  | - | - | 119  (102, 178) | Intervention evaluated against early diagnosis and prompt treatment |
|  |  |  | |  |  |  |  |  |  |
| Kamolratanakul 2001 | Thai-Myanmar border  1993 – 1994 | Whole villages – IRS (DDT) | | Provider | incidence > 0.2 | - | - | 2.78 |  |
|  |  |  | |  |  |  |  |  |  |
| Worrall  2008 | Zimbabwe  1993 – 1998 | Whole population – IRS 24% coverage | | Provider | variable | - | - | 0.54 – 140 | This study investigates how to use a Malaria Early Warning System in conjunction with IRS. The lower estimate is for a year when there is a malaria epidemic, the higher estimate for a year when there is very little malaria. |
|  |  | Whole population – IRS 24% coverage | |  |  | - | - | 0.93 – 267 |  |
|  |  |  | |  |  |  |  |  |  |
| Yukich  2008 | KwaZulu-Natal, SA 1997-1999 | Pyrethroid, yearly | | Provider |  | 4961 | 150 | - | This result is based on data from Goodman *et al* . Costs are economic costs. |
|  |  |  | |  |  |  |  |  |  |
| Yukich  2008 | Mozambique 1999-2001 | Children 2-15years (Ficam, Propoxur) | | Provider | seasonal | 4478 | 135 | - | This result is based on data from the study by Conteh *et al* also referenced below. Costs are economic costs. |
|  |  |  | |  |  |  |  |  |  |
| Conteh  2004 | Mozambique  1999 – 2000 | Children  2-15years – rural IRS (Ficam, Propoxur) | | Provider | *Pf*PR_2-15_=  0.64-0.86 | - | - | 27  (11.60, 32) | Estimate for rural area is per clinical case. 2000 US$21.23 per infection averted. CE estimates not given for the peri-urban area. |
|  |  | Children  2-15years – peri-urban IRS (Ficam, Propoxur) | |  |  | - | - | - |  |
|  |  |  |  |  |  |  |  |  |  |
| Guyatt  2002 | Kenyan highlands  1999 – 2000 | Whole population – IRS (lambdacyhalothrin) | | Provider | epidemic | - | - | 11.62 | This study was undertaken during an epidemic. Cases are infections averted. |
|  |  |  | |  |  |  |  |  |  |
| **Intermittent Preventive Treatment** | | | | | |  |  |  |  |
| Sicuri 2010 | southern Mozambique | IPTp (SP) – pregnant women – ANC delivery with ITN | Society – maternal effect | |  | - | 44.24 (21.87, 103.18) | - | There is the potential to extend to costs per case and death averted if the details in Sicuri *et al*’s Table 2 can be worked out with the correct model assumptions. |
|  |  | IPTp (SP) – pregnant women – ANC delivery with ITN | Society – neonatal effect | |  | - | 1.15  (0.45, 3.71) | - |  |
|  |  | IPTp (SP) – pregnant women – ANC delivery with ITN | Society – total effect | |  | - | 1.08  (0.44, 3.42) | - |  |
|  |  |  |  | |  |  |  |  |  |
| Mbonye 2008 | Uganda | IPTp (SP 2 dose) pregnant women – community | Society | |  | - | 1.28  (0.63, 17.31) | - | This study calculates the incremental cost effectiveness of IPT delivered through the community over health centre IPT. ICERs are not relative to a baseline with no intervention and so comparisons cannot be made with other studies. |
|  |  |  |  | |  |  |  |  |  |
| Wolfe 2001 | Sub-Saharan Africa | IPTp (2 dose SP) – pregnant women – ANC | Provider | |  | - | 11 | - | This is a model based study based on cohort of 10,000 pregnant women with high HIV prevalence (27%). DALYs gained accrue to infants due to LBW aversion. |
|  |  | IPTp (monthly dose SP) – pregnant women – ANC |  | |  | - | 14 | - |  |
|  |  |  |  | |  |  |  |  |  |
| Goodman 2001 | Sub-Saharan Africa | IPTp (2 dose SP) – pregnant women – ANC | Provider – low income | |  | - | 17.43  (5.81, 39.23) | - | Estimates are for primigravidae. |
|  |  | IPTp (2 dose SP) – pregnant women – ANC | Provider – mid income | |  | - | 18.89  (5.81, 42.13) | - |  |
|  |  | IPTp (2 dose SP) – pregnant women – ANC | Provider – high income | |  | - | 29.06  (10.17, 62.48) | - |  |
|  |  | IPTp (2 dose CQ) – pregnant women –ANC | Provider – low income | |  | - | 30.51  (10.17, 71.20) | - |  |
|  |  | IPTp (2 dose CQ) – pregnant women – ANC | Provider – mid income | |  | - | 33.42  (11.62, 75.56) | - |  |
|  |  |  |  | |  |  |  |  |  |
|  |  | IPTp (2 dose CQ) – pregnant women – ANC | Provider – high income | |  | - | 49.40 (17.43, 108.98) | - |  |
|  |  |  |  | |  |  |  |  |  |
| Conteh  2010 | Hohoe, Ghana  2005 | IPTc (monthly SP) children 3 – 59 months | Provider | | Seasonal  (EIR = 65) | - | - | 29  (21, 44) | The costs presented here are for IPTc scaled up to a district level. Other cost-effectiveness ratios presented in the original publication are based on a small well-organised trial with high trial-associated expenses and so wouldn’t be comparable to large-scale deployment of interventions. |
|  |  | IPTc (bimonthly AS & AQ) children 3 – 59 months |  | |  | - | - | 23  (21, 25) |  |
|  |  | IPTc (monthly AS & AQ) children AS & AQ |  | |  | - | - | 62  (38, 118) |  |
|  |  |  |  | |  |  |  |  |  |
| Temperley 2008 | Western Kenya  2005 – 2006 | IPTc (SP+AQ) – anaemia in school age children | Provider | | Stable and perennial | - | - | 33  (27, 44) | This study uses endpoints of cases of anaemia or parasitaemia averted as opposed to uncomplicated or severe episodes averted. |
|  |  | IPTc (SP+AQ) – parasitaemia in school age children |  | |  | - | - | 5.90  (4.62, 13.03) |  |
|  |  |  |  | |  |  |  |  |  |
|  |  |  |  | |  |  |  |  |  |
| Picard  1992 | Gambia | IPTc (Maloprim) – children  3-59 months | Provider | |  | 248 | - | - | This trial was carried out before the term IPT was coined, and hence the intervention is referred to as chemoprophylaxis in the paper. |
|  |  |  |  | |  |  |  |  |  |
| Conteh  2010 | Ifakara, Tanzania  1999 – 2001 | IPTi (SP) – EPI | Society | | Perennial  (EIR = 29) | - | 3.09  (1.71, 5.01) | 1.45  (0.96, 2.09) | The cost-effectiveness ratios are taken from Conteh *et* al’s Table S4. For SP the trial results and not the pooled results are taken. The negative cost-effectiveness results for Same, Tanzania are because incidence of malaria went up by chance in the group given IPTi, possibly because of the very low baseline level of transmission. As such, care should be taken when comparing these results with those from other studies. |
|  | Navrongo, Ghana  2000 – 2004 | IPTi (SP) – EPI | Society | | Highly seasonal (EIR = 418) | - | 1.60  (0.96, 2.55) | 3.25  (1.66, 5.67) |  |
|  | Manhiça, Mozambique  2002 – 2004 | IPTi (SP) – EPI | Society | | Perennial with seasonal peaks (EIR = 38) | - | 4.39  (1.82, 9.79) | 9.20  (3.43, 21.31) |  |
|  | Kumasi, Ghana  2003 – 2005 | IPTi (SP) – EPI | Society | | Perennial with seasonal peaks (EIR = 400) | - | 1.77  (1.02, 2.91) | 3.61  (1.79, 6.56) |  |
|  | Tamale, Ghana  2003 – 2005 | IPTi (SP) – EPI | Society | | Perennial with seasonal peaks (EIR NA) | - | 1.65  (0.97, 2.58) | 3.37  (1.69, 5.95) |  |
|  | Lambaréné, Gabon  2002 – 2006 | IPTi (SP) – EPI | Society | | Perennial with seasonal peaks (EIR = 50) | - | 13  (-96, 109) | 31  (-197, 223) |  |
|  | Korogwe, Tanzania  2004 – 2008 | IPTi (SP) – EPI | Society | | Perennial with seasonal peak (EIR NA) | - | -17  (-64, 57) | -40  (-140, 119) |  |
|  | Same, Tanzania  2004 – 2008 | IPTi (SP) – EPI | Society | | Low  (EIR NA) | - | -0.75  (-77, 3.83) | -7.83  (-165,-7.28) |  |
|  | Western Kenya  2004 – 2007 | IPTi (SP-AS3) – EPI | Society | | Perennial  (EIR = 7) | - | 8.23  (3.67,18) | 17.16 (6.69,39) |  |
|  | Western Kenya  2004 – 2007 | IPTi (AQ3-AS3) – EPI | Society | | Perennial  (EIR = 7) | - | 4.92  (2.46, 9.46) | 10.27  (4.49, 21) |  |
|  | Western Kenya  2004 – 2007 | IPTi (CD3) – EPI | Society | | Perennial  (EIR = 7) | - | 12  (-163, 84) | 42  (-376, 411) |  |
|  | Korogwe,Tanzania  2004 – 2008 | IPTi (MQ) – EPI | Society | | Perennial with seasonal peak (EIR NA) | - | 20  (10, 36) | 42  (19, 82) |  |
|  | Korogwe,Tanzania  2004 – 2008 | IPTi (CD3) – EPI | Society | | Perennial with seasonal peak (EIR NA) | - | 139  (-641, 714) | 260  (-1406, 447) |  |
|  | Same,Tanzania  2004 – 2008 | IPTi (MQ) – EPI | Society | | Low  (EIR NA) | - | -225  (-1124, 1242) | -294  (-2473, 2620) |  |
|  | Same,Tanzania  2004 – 2008 | IPTi (CD3) – EPI | Society | | Low  (EIR NA) | - | -329  (-1166, 681) | -676  (-2454, 1338) |  |
|  |  |  |  | |  |  |  |  |  |
| Hutton  2009 | Ifakara, Tanzania  1999 – 2001 | IPTi (SP) – EPI | Society | | Perennial  (EIR = 29) | 110  (47, 364) | 4.07  (1.76, 13.43) | 1.76  (0.88, 4.40) | The data used for this study is the same also used in the study by Conteh *et al* but the analysis is different. |
|  | Manhiça, Mozambique  2002 – 2004 | IPTi (SP) – EPI | Society | | Perennial with seasonal peaks (EIR = 38) | 332  (105, 2751) | 12.33  (3.96, 101) | 5.17  (1.87, 33) |  |
|  |  |  |  |  |  |  |  |  |  |
| **Vaccines** | | | |  |  |  |  |  |  |
| Tediosi  2009 | Tanzania | Vaccine – EPI delivery to children | | Society | EIR = 21 | 1163 – 5362 | 38 – 176 | 5.83 – 26 | Vaccine based on RTS,S. 52% infection-blocking efficacy. EPI delivery over 20 years. Price per dose varying from 2006 USD 2-10. This study presents a wide range of estimates of the cost-effectiveness of vaccines in different transmission sites and at different costs. Only a subset of the results are shown here. Please see the original publication for more details. |
|  |  |  | |  |  |  |  |  |  |
| Tediosi  2006 | Tanzania  2004 | Vaccine – EPI delivery to children | | Society | EIR = 21 | 443 – 7049 | 13 – 213 | 3.50 – 61 | Vaccine based on RTS,S. 52% infection-blocking efficacy. EPI delivery over 5 years with EIR=21. Costs are average costs. Price per dose varying from 2004 US$1-20 |
|  |  |  | |  |  |  |  |  |  |
| Graves  1998 | The Gambia  1990 | Vaccine – children 0-4 years | | Provider | incidence = 1 episode per year | 437  (284, 592) | - | 6.44  (4.18, 8.70) | Vaccine causes 39% reduction in clinical attacks and 20% reduction in malaria-associated deaths. 1990 US$ 4.29 per fully immunised child. |
|  |  |  |  |  |  |  |  |  |  |
| WHO  1996 | Sub-Saharan Africa, 1990 | Vaccine – EPI delivery to children 0-4 years | | Provider | high mortality region | - | 0.69 – 19.10 | - | 30% reduction in all-cause mortality in children < 5 years. EPI delivery. Protection lasts 1-5 years, cost between 1990 US$1 and 1990 US$7.50 per child per year.  Same as above except no EPI delivery, protection lasts 1 year, costs 1990 US$15 per child per year |
|  |  |  | |  | low mortality region | - | 9.81 – 276 | - |  |
|  |  | Vaccine – one-off delivery to children 0-4 years | |  | high mortality region | - | 41 | - |  |
|  |  |  | |  | low mortality region | - | 601 | - |  |
|  |  |  | |  |  |  |  |  |  |
| **Environmental Management** | | | |  |  |  |  |  |  |
| Utzinger  2001 | Zambia  1930 – 1949 | Whole population – environmental management (entire period) | | Provider | *Pf*PR = 0.26  (pre-intervention) | 1246 | - | 32 | Environmental management consists of: vegetation clearance along the river and its tributaries; modification of river boundaries and removal of manmade obstruction; and draining flooded areas and swamps. Study was conducted at a copper mine, |
|  |  | Whole population – environmental management (maintenance) | |  |  | - | 32 – 133 | - |  |
|  |  | Whole population – environmental management ( 5 year start up) | |  |  | - | 762 | - |  |
| ***Treatment*** | | | |  |  |  |  |  |  |
| **Drug treatment** | |  | |  |  |  |  |  |  |
| Lubell  2011 | Teule, Tanzania  2009 – 2010 | Artesunate for severe malaria in children – hospital inpatients | | Provider | high, seasonal | 49 | 1.50 | 1.2 | Results presented are ICERs for treatment of severe malaria with artesunate compared to treatment with quinine. The results for Korogwe appear negative because it was both cheaper and more efficacious to treat with ART.  The pooled results are averaged across the 4 sites. |
|  | Korogwe, Tanzania  2009 – 2010 |  |  | Provider | low, seasonal | -66 | -2.00 | -1.60 |  |
|  | Mbarara, Uganda  2009 – 2010 |  |  | Provider | high, seasonal | 41 | 1.20 | 1.00 |  |
|  | Ilorin, Nigeria  2009 – 2010 |  |  | Provider | high, seasonal | 922 | 28.10 | 22.50 |  |
|  | Pooled SSA  2009 – 2010 |  |  | Provider |  | 123 | 3.80 | 3.00 |  |
|  |  |  | |  |  |  |  |  |  |
| Davis  2011 | Papua New Guinea  2005 – 2007 | ART & SP for treatment of uncomplicated *P. falciparum* in children | | Society |  | - | - | 10.59 | The results presented here are ICERs for cases of uncomplicated malaria treated using artemisinin therapies compared to a baseline of conventional treatment with CQ & SP. The sensitivity analyses presented in Table 3 of Davis *et al* appears to give contradictory results and hence is not included here.  The negative result for treatment of *P. vivax* with DHA & PQ is because it was both cheaper and more effective than conventional treatment. |
|  |  | DHA & PQ for treatment of uncomplicated *P. falciparum* in children | | Society |  | - | - | 3.06 |  |
|  |  | AL for treatment of uncomplicated *P. falciparum* in children | | Society |  | - | - | 7.23 |  |
|  |  | ART & SP for treatment of uncomplicated *P. vivax* in children | | Society |  | - | - | 2.83 |  |
|  |  | ART & SP for treatment of uncomplicated *P. vivax* in children | | Society |  | - | - | -0.18 |  |
|  |  | ART & SP for treatment of uncomplicated *P. vivax* in children | | Society |  | - | - | 6.95 |  |
|  |  |  | |  |  |  |  |  |  |
| Buchanan  2010 | Sub-Saharan Africa | rectal anti-malarial for severe malaria in children < 5years | | Provider |  | 124 | 3.41 | - | This is a model based study of rectally administered anti-malarials for the treatment of severe malaria. ICERs are calculated against a usual practice scenario where no rectal treatments for severe febrile illness are widely used. |
|  | Sub-Saharan Africa | rectal anti-malarial for severe malaria 5 years and older | | Provider |  | 253 | 13.66 | - |  |
|  | Sub-Saharan Africa | rectal anti-malarial for severe malaria in all ages | | Provider |  | 169 | 5.69 | - |  |
|  | South-east Asia | rectal anti-malarial for severe malaria in children < 5years | | Provider |  | 13255 | 392 | - |  |
|  | South-east Asia | rectal anti-malarial for severe malaria 5 years and older | | Provider |  | 4772 | 202 | - |  |
|  | South-east Asia | rectal anti-malarial for severe malaria in all ages | | Provider |  | 5043 | 202 | - |  |
|  |  |  | |  |  |  |  |  |  |
| Tozan  2010 | Sub-Saharan Africa | rectal artesunate for treatment of severe malaria in a cohort of 1000 children over 5 years | | Provider |  | - | 141  (80 – 1218) | - | This model is based on a simulated cohort of 1000 children over 5 years using data from an RCT trial. Tozan *et al*’s Table 1 ICERs are given for a range of compliance and uptake. We take 50% compliance and 50% uptake and use the other figures for sensitivity analyses. The baseline scenario for ICER comparison is no treatment. |
|  |  |  | |  |  |  |  |  |  |
| Lubell  2009 | SE Asia  2003 – 2005 | ACT for severe malaria | |  |  | 141 | - | - | This ICER is for a pooled analysis. ICERs for single study areas are US$ 184 in Bangladesh, US$ 373 in India, US$ 193 in Indonesia, and US$ 109 in Myanmar. |
|  |  |  | |  |  |  |  |  |  |
| Chanda  2007 | Zambia  2004 | Patients at public health facilities ACT vs. SP (first line) | | Provider | incidence = 0.42 | - | - | 4.66 | Cost-effectiveness of artemether-lumefantrine vs. SP. |
|  |  | Patients at public health facilities ACT vs. SP (first + second line) | |  |  | - | - | -13.11 |  |
|  |  | Patients at public health facilities ACT (first line) | |  |  | - | - | 9.75 | Cost-effectiveness of AL versus no treatment. |
|  |  | Patients at public health facilities ACT (first + second line) | |  |  | - | - | 11.29 |  |
|  |  |  | |  |  |  |  |  |  |
| Wiseman 2006 | Tanga, Tanzania  2002 – 2004 | Children (AQ+SP vs. AQ) | | Provider | EIR > 300 | - | - | 0.25 | Interventions evaluated against a baseline of AQ monotherapy at day 14. |
|  |  | Children (AQ+AS vs. AQ) | |  |  | - |  | -0.03 |  |
|  |  | Children (AL vs. AQ) | |  |  | - | - | -0.2 |  |
|  |  | Children (AQ+SP vs. AQ) | | Society |  | - | - | -24.53 |  |
|  |  | Children (AQ+AS vs. AQ) | |  |  | - | - | -26.77 |  |
|  |  | Children (AL vs. AQ) | |  |  | - | - | -27.03 |  |
|  |  |  | |  |  |  |  |  |  |
|  |  |  | |  |  |  |  |  |  |
|  |  |  | |  |  |  |  |  |  |
| Coleman 2004 | Sub-Saharan Africa | Whole population – ACT (30% initial SP resistance) | | Provider |  | - | 37 | - | The results of this study are not directly comparable with other studies as ACT resistance is assumed to be emerging. Cost-effectiveness evaluated over a 5 year window. |
|  |  | Whole population – ACT (70% initial SP resistance) | |  |  | - | 13.01 | - |  |
| Honrado 1999 | Thailand | Quinine + tetracycline | |  |  | - | - | 28 | This is an old formulation of artesunate and may not be relevant for modern ACTs. |
|  |  | artesunate | |  |  | - | - | 25 |  |
|  | |  | |  |  |  |  |  |  |
| **Diagnostics** | |  | |  |  |  |  |  |  |
| Chanda  2011 | Zambia 2009 | Comparison of diagnosis and treatment with HMM compared to CHW | | Provider | Moderate  *Pf*PR = 0.27 | - | - | 4.18 | This is a comparison of the savings that can be made by diagnosing and treating with Home Management of Malaria (HMM) compared Community Health Workers (CHW) at health facilities. |
|  |  |  | |  |  |  |  |  |  |
| Lemma  2011 | Ethiopia 2007 | Parascreen – RDT & AL/CQ and referral otherwise | | Provider | seasonal, unstable | - | - | 0.62 | ICERs are compared to the Paracheck strategy – use of *P. falciparum* specific RDT and AL for *P. falciparum* cases and CQ for the rest |
|  |  | Presumptive treatment with AL | | Provider | seasonal, unstable | - | - | -0.87 | Presumptive treatment is the dominant strategy. |
|  |  |  | |  |  |  |  |  |  |
| Uzochukwu 2009 | Nigeria  2005 – 2007 | People presenting to public health facility – RDT with ACT | | Society | *Pf*PR = 0.43 | -243 | - | - | This is a modelling study comparing RDTs and microscopy against a baseline of presumptive treatment. RDTs were cheaper than presumptive treatment.. |
|  |  | People presenting to public health facility – microscopy with ACT | |  |  | 283 | - | - |  |
|  |  |  | |  |  |  |  |  |  |
| ***Combined treatment and prevention*** | | | | |  |  |  |  |  |
| Akhavan 1999 | Amazon Basin, Brazil  1988 – 1996 | Whole population - multiple interventions | | Provider | incidence =  0.01 – 0.03 | 3522  (1409, 7045) | 90 | - | Savings on treatment are incorporated into estimates. Interventions consisted of vector control (IRS and fogging), environmental management, treatment, surveillance and  education. |
|  |  | Whole population – prevention only | |  |  | 6796  (2718, 13592) | 177 | - |  |
|  |  | Whole population – treatment only | |  |  | 985  (394, 1970) | 25 | - |  |
| Mills  1993 | Nepal | Whole population - multiple interventions | |  |  | 158 – 25647 | 17 – 2620 | - | Interventions are case detection and treatment, and IRS. |

**References**

1. Bhatia MR, Fox-Rushby J, Mills A: **Cost-effectiveness of malaria control interventions when malaria mortality is low: insecticide-treated nets versus in-house residual spraying in India**. *Social Science & Medicine* 2004, **59**(3):525-539.

2. Kamolratanakul P, Butraporn P, Prasittisuk M, Prasittisuk C, Indaratna K: **Cost-effectiveness and sustainability of lambdacyhalothrin-treated mosquito nets in comparison to DDT spraying for malaria control in western Thailand**. *American Journal of Tropical Medicine and Hygiene* 2001, **65**(4):279-284.

3. Becker-Dreps SI, Biddle AK, Pettifor A, Musuamba G, Imbie DN, Meshnick S, Behets F: **Cost-effectiveness of adding bed net distribution for malaria prevention to antenatal services in Kinshasa, Democratic Republic of the Congo**. *American Journal of Tropical Medicine and Hygiene* 2009, **81**(3):496-502.

4. Yukich JO, Zerom M, Ghebremeskel T, Tediosi F, Lengeler C: **Costs and cost-effectiveness of vector control in Eritrea using insecticide-treated bed nets**. *Malaria Journal* 2009, **8**.

5. Mueller DH, Wiseman V, Bakusa D, Morgah K, Dare A, Tchamdja P: **Cost-effectiveness analysis of insecticide-treated net distribution as part of the Togo Integrated Child Health Campaign**. *Malaria Journal* 2008, **7**.

6. Yukich JO, Lengeler C, Tediosi F, Brown N, Mulligan J-A, Chavasse D, Stevens W, Justino J, Conteh L, Maharaj R *et al*: **Costs and consequences of large-scale vector control for malaria**. *Malaria Journal* 2008, **7**.

7. Wiseman V, Hawley WA, ter Kuile FO, Phillips-Howard PA, Vulule JM, Nahlen BL, Mills AJ: **The cost-effectiveness of permethrin-treated bed nets in an area of intense malaria transmission in western Kenya**. *American Journal of Tropical Medicine and Hygiene* 2003, **68**(4):161-167.

8. Hanson K, Kikumbih N, Schellenberg JA, Mponda H, Nathan R, Lake S, Mills A, Tanner M, Lengeler C: **Cost-effectiveness of social marketing of insecticide-treated nets for malaria control in the United Republic of Tanzania**. *Bulletin of the World Health Organization* 2003, **81**(4):269-276.

9. Guyatt HL, Corlett SK, Robinson TP, Ochola SA, Snow RW: **Malaria prevention in highland Kenya: indoor residual house-spraying vs. insecticide-treated bednets**. *Tropical Medicine & International Health* 2002, **7**(4):298-303.

10. Goodman CA, Mnzava AEP, Dlamini SS, Sharp BL, Mthembu DJ, Gumede JK: **Comparison of the cost and cost-effectiveness of insecticide-treated bednets and residual house-spraying in KwaZulu-Natal, South Africa**. *Tropical Medicine & International Health* 2001, **6**(4):280-295.

11. Coleman PG, Goodman CA, Mills A: **Rebound mortality and the cost-effectiveness of malaria control: potential impact of increased mortality in late childhood following the introduction of insecticide treated nets**. *Tropical Medicine & International Health* 1999, **4**(3):175-186.

12. Some E: **Optimizing the community effectiveness of insecticide-impregnated bednets used for malaria control in coastal Kenya: Implications of perceptions, programme organization, compliance, and costs.** *PhD Thesis* 1999.

13. Guyatt HL, Snow RW, Evans DB: **Malaria epidemiology and economics: the effect of delayed immune acquisition on the cost-effectiveness of insecticide-treated bednets**. *Philosophical Transactions of the Royal Society of London Series B-Biological Sciences* 1999, **354**(1384):827-835.

14. Graves PM: **Comparison of the cost-effectiveness of vaccines and insecticide impregnation of mosquito nets for the prevention of malaria**. *Annals of Tropical Medicine and Parasitology* 1998, **92**(4):399-410.

15. Aikins MK, Fox-Rushby J, D'Alessandro U, Langerock P, Cham K, New L, Bennett S, Greenwood B, Mills A: **The Gambian National Impregnated Bednet Programme: Costs, consequences and net cost-effectiveness**. *Social Science & Medicine* 1998, **46**(2):181-191.

16. Binka FN, Mensah OA, Mills A: **The cost-effectiveness of permethrin impregnated bednets in preventing child mortality in Kassena-Nankana district of northern Ghana**. *Health Policy* 1997, **41**(3):229-239.

17. Evans DB, Azene G, Kirigia J: **Should governments subsidize the use of insecticide-impregnated mosquito nets in Africa? Implications of a cost-effectiveness analysis**. *Health Policy and Planning* 1997, **12**(2):107-114.

18. WHO: **Investing in Health Research and Development: Report of the Ad Hoc Committee on Health Relating to Future Intervention Options**, vol. TDR/Gen/96.1. Geneva; 1996.

19. Picard J, Aikins M, Alonso PL, Schellenberg J, Greenwood BM, Mills A: **A MALARIA CONTROL TRIAL USING INSECTICIDE-TREATED BED NETS AND TARGETED CHEMOPROPHYLAXIS IN A RURAL AREA OF THE GAMBIA, WEST-AFRICA .8. COST-EFFECTIVENESS OF BED NET IMPREGNATION ALONE OR COMBINED WITH CHEMOPROPHYLAXIS IN PREVENTING MORTALITY AND MORBIDITY FROM MALARIA IN GAMBIAN CHILDREN**. *Transactions of the Royal Society of Tropical Medicine and Hygiene* 1993, **87**:53-57.

20. Worrall E, Connor SJ, Thomson MC: **Improving the cost-effectiveness of IRS with climate informed health surveillance systems**. *Malaria Journal* 2008, **7**.

21. Conteh L, Sharp BL, Streat E, Barreto A, Konar S: **The cost and cost-effectiveness of malaria vector control by residual insecticide house-spraying in southern Mozambique: a rural and urban analysis**. *Tropical Medicine & International Health* 2004, **9**(1):125-132.

22. Conteh L, Patouillard, E, Kweku, M, Legood, R, Greenwood, B, Chandramohan, D,: **Cost Effectiveness of Seasonal Intermittent Preventive Treatment using Amodiaquine & Artesunate or Sulphadoxine-Pyrimethamine in Ghanaian Children**. *PLoS One* 2010.

23. Temperley M, Mueller DH, Njagi JK, Akhwale W, Clarke SE, Jukes MCH, Estambale BBA, Brooker S: **Costs and cost-effectiveness of delivering intermittent preventive treatment through schools in western Kenya**. *Malaria Journal* 2008, **7**.

24. Picard J, Mills A, Greenwood B: **THE COST-EFFECTIVENESS OF CHEMOPROPHYLAXIS WITH MALOPRIM(R) ADMINISTERED BY PRIMARY HEALTH-CARE WORKERS IN PREVENTING DEATH FROM MALARIA AMONGST RURAL GAMBIAN CHILDREN AGED LESS THAN 5 YEARS OLD**. *Transactions of the Royal Society of Tropical Medicine and Hygiene* 1992, **86**(6):580-581.

25. Conteh L, Sicuri E, Manzi F, Hutton G, Obonyo B, Tediosi F, Biao P, Masika P, Matovu F, Otieno P *et al*: **The cost-effectiveness of intermittent preventive treatment for malaria in infants in Sub-Saharan Africa**. *PLoS One* 2010, **5**(6):e10313.

26. Hutton G, Schellenberg D, Tediosi F, Macete E, Kahigwa E, Sigauque B, Mas X, Trapero M, Tanner M, Trilla A *et al*: **Cost-effectiveness of malaria intermittent preventive treatment in infants (IPTi) in Mozambique and the United Republic of Tanzania**. *Bulletin of the World Health Organization* 2009, **87**(2):123-129.

27. Tediosi F, Maire N, Penny M, Studer A, Smith TA: **Simulation of the cost-effectiveness of malaria vaccines**. *Malaria Journal* 2009, **8**.

28. Tediosi F, Hutton G, Maire N, Smith TA, Ross A, Tanner M: **Predicting the cost-effectiveness of introducing a pre-erythrocytic malaria vaccine into the expanded program on immunization in Tanzania**. *American Journal of Tropical Medicine and Hygiene* 2006, **75**(2):131-143.

29. Utzinger J, Tozan Y, Singer BH: **Efficacy and cost-effectiveness of environmental management for malaria control**. *Tropical Medicine & International Health* 2001, **6**(9):677-687.

30. Lubell Y, Riewpaiboon A, Dondorp AM, von Seidlein L, Mokuolu OA, Nansumba M, Gesase S, Kent A, Mtove G, Olaosebikan R *et al*: **Cost-effectiveness of parenteral artesunate for treating children with severe malaria in sub-Saharan Africa**. *Bulletin of the World Health Organization* 2011, **89**(7):504-512.

31. Davis WA, Clarke PM, Siba PM, Karunajeewa HA, Davy C, Mueller I, Davis TME: **Cost-effectiveness of artemisinin combination therapy for uncomplicated malaria in children: data from Papua New Guinea**. *Bulletin of the World Health Organization* 2011, **89**(3):211-220.

32. Buchanan J, Mihaylova B, Gray A, White N: **Cost-Effectiveness of Pre-Referral Antimalarial, Antibacterial, and Combined Rectal Formulations for Severe Febrile Illness**. *Plos One* 2010, **5**(12).

33. Tozan Y, Klein EY, Darley S, Panicker R, Laxminarayan R, Breman JG: **Prereferral rectal artesunate for treatment of severe childhood malaria: a cost-effectiveness analysis**. *Lancet* 2010, **376**(9756):1910-1915.

34. Lubell Y, Yeung S, Dondorp AM, Day NP, Nosten F, Tjitra E, Faiz MA, Bin Yunus E, Anstey NM, Mishra SK *et al*: **Cost-effectiveness of artesunate for the treatment of severe malaria**. *Tropical Medicine & International Health* 2009, **14**(3):332-337.

35. Chanda P, Masiye F, Chitah BM, Sipilanyambe N, Hawela M, Banda P, Okorosobo T: **A cost-effectiveness analysis of artemether lumefantrine for treatment of uncomplicated malaria in Zambia**. *Malaria Journal* 2007, **6**.

36. Wiseman V, Kim M, Mutabingwa TK, Whitty CJM: **Cost-effectiveness study of three antimalarial drug combinations in Tanzania**. *Plos Medicine* 2006, **3**:1844-1850.

37. Coleman PG, Morel C, Shillcutt S, Goodman C, Mills AJ: **A threshold analysis of the cost-effectiveness of artemisinin-based combination therapies in sub-Saharan Africa**. *American Journal of Tropical Medicine and Hygiene* 2004, **71**(2):196-204.

38. Honrado ER, Fungladda W, Kamoiratanaku P, Kitayaporn D, Karbwang J, Thimasarn K, Masngammueng R: **Cost-effectiveness analysis of artesunate and quinine plus tetracycline for the treatment of uncomplicated falciparum malaria in Chanthaburi, Thailand**. *Bulletin of the World Health Organization* 1999, **77**(3):235-243.

39. Chanda P, Hamainza B, Moonga HB, Chalwe V, Banda P, Pagnoni F: **Relative costs and effectiveness of treating uncomplicated malaria in two rural districts in Zambia: implications for nationwide scale-up of home-based management**. *Malaria Journal* 2011, **10**.

40. Lemma H, San Sebastian M, Lofgren C, Barnabas G: **Cost-effectiveness of three malaria treatment strategies in rural Tigray, Ethiopia where both Plasmodium falciparum and Plasmodium vivax co-dominate**. *Cost effectiveness and resource allocation : C/E* 2011, **9**:2.

41. Uzochukwu BSC, Obikeze EN, Onwujekwe OE, Onoka CA, Griffiths UK: **Cost-effectiveness analysis of rapid diagnostic test, microscopy and syndromic approach in the diagnosis of malaria in Nigeria: implications for scaling-up deployment of ACT**. *Malaria Journal* 2009, **8**.

42. Akhavan D, Musgrove P, Abrantes A, Gusmao RD: **Cost-effective malaria control in Brazil - Cost-effectiveness of a Malaria Control Program in the Amazon Basin of Brazil, 1988-1996**. *Social Science & Medicine* 1999, **49**(10):1385-1399.

43. Mills A: **Is malaria control a priority? Evidence from Nepal**. *Health Econ* 1993, **2**(4):333-347.
